# Outcomes in Patients With Atrial Fibrillation Stratified by Body Mass Index and Heart Failure Status

**DOI:** 10.1016/j.jacadv.2025.102531

**Published:** 2026-01-19

**Authors:** Jean Jacques Noubiap, Lisa A. Kaltenbach, Karen Chiswell, Ulrich Flore Nyaga, Mina K. Chung, Jeroen M. Hendriks, Larry R. Jackson, Andrea M. Russo, Annabelle Santos Volgman, Meghan Reading Turchioe, Ohad Ziv, Jonathan P. Piccini, Prashanthan Sanders

**Affiliations:** a Centre for Heart Rhythm Disorders, Adelaide University and Royal Adelaide Hospital, Adelaide, Australia; b Division of Cardiology, Department of Medicine, University of California-San Francisco, San Francisco, California, USA; c Duke Clinical Research Institute, Duke University Medical Center, Durham, North Carolina, USA; d Health Data Acumen, Nairobi, Kenya; e Department of Medicine, Edea Regional Hospital, Edea, Cameroon; f Department of Cardiovascular Medicine, Heart, Vascular & Thoracic Institute, Cleveland Clinic, Cleveland, USA; g Department of Nursing, Maastricht University Medical Centre, Maastricht, The Netherlands; h Department of Health Services Research, Care and Public Health Research Institute, Maastricht University, Maastricht, The Netherlands; i Department of Medicine, Cooper Medical School of Rowan University, Camden, New Jersey, USA; j Division of Cardiology, Rush University Medical Center, Chicago, Illinois, USA; k School of Nursing, Columbia University School of Nursing, New York, New York, USA; l Heart and Vascular Research Center, MetroHealth Campus, Case Western Reserve University, Cleveland, Ohio, USA

**Keywords:** atrial fibrillation, heart failure, mortality, myocardial infarction, obesity, stroke

## Abstract

**Background:**

An obesity-survival benefit, called the “obesity paradox,” has been variably reported in patients with heart failure (HF) and those with atrial fibrillation (AF), but inconsistencies have been observed.

**Objectives:**

The purpose of this study was to assess how the interaction between body mass index (BMI) and HF status impacts AF-related outcomes.

**Methods:**

Patients hospitalized for AF in the Get With The Guidelines-Atrial Fibrillation registry from 2013 to 2021 and linked to Medicare claims were included. Adjusted Cox proportional hazards models were used to assess the association between BMI and outcomes, stratified by HF status (no HF, HF with preserved ejection fraction, HF with mid-range ejection fraction, and HF with reduced ejection fraction). The outcomes were mortality, cardiovascular rehospitalization, thromboembolism, and myocardial infarction within 1 year.

**Results:**

In total, 21,850 patients (mean age 77 years, 42.3% male) were included: 29.5% underweight/normal (BMI<25 kg/m^2^), 32.0% overweight (BMI 25-29.9), and 38.5% obese (BMI ≥30). Increasing BMI was associated with lower mortality in patients without obesity and without HF (HR: 0.94 per 1 kg/m^2^ increase; 95% CI: 0.92 to 0.95), with HF with reduced ejection fraction (HR: 0.96; 0.93-0.99), and with HF with preserved ejection fraction/HF with mid-range ejection fraction (HR: 0.93; 0.92-0.95), while increases in BMI among patients with obesity were not associated with lower mortality (interaction *P* = 0.013). The risks of cardiovascular rehospitalization, thromboembolism, and myocardial infarction were not significantly different across the HF spectrum between patients with and without obesity (all interaction *P* > 0.05).

**Conclusions:**

Higher BMI was associated with increased survival in patients without obesity, irrespective of HF status, but not in patients with obesity and AF.

Although obesity is a well-established risk factor for the development of heart failure (HF),^1^, ^2^, ^3^, ^4^ several studies have reported counterintuitive findings suggesting that, among people with chronic HF, overweight and mild to moderate obesity are associated with substantially lower mortality compared with normal weight.^5^, ^6^, ^7^, ^8^ This “obesity paradox” has been mostly described in patients with HF with reduced ejection fraction (HFrEF), whereas it was inconsistently observed in those with HF with preserved ejection fraction (HFpEF).^5^^,^^6^ The majority of studies that described the “obesity paradox” in HF measured obesity by body mass index (BMI), and studies that used other surrogates of adiposity confirmed this obesity paradox, but with some discrepancies.^8^, ^9^, ^10^, ^11^

Obesity is commonly seen in patients with atrial fibrillation (AF), a prevalent arrhythmia^12^ associated with increased risk of ischemic stroke,^13^ ischemic heart disease,^14^ and premature death.^15^ Obesity is an important driver of the occurrence and progression of AF,^16^, ^17^, ^18^, ^19^ but its prognostic implications in patients with AF are complex. Long-term sustained weight loss has been shown to be associated with a significant reduction of AF burden and maintenance of sinus rhythm,^17^, ^18^, ^19^, ^20^, ^21^, ^22^, ^23^ prompting the inclusion of weight reduction and other risk factor control as a new pillar of AF management.^18^^,^^19^ However, the impact of obesity on hard outcomes, such as death or ischemic stroke, in patients with AF is unclear. Randomized controlled trials (RCTs) of oral anticoagulation showed lower risks of ischemic stroke and mortality associated with obesity,^24^, ^25^, ^26^, ^27^ supporting an “obesity paradox.” Conversely, observational studies mostly showed either increased mortality or no impact on mortality and ischemic stroke of obesity compared with normal weight.^27^, ^28^, ^29^

To our knowledge, the influence of HF on the relationship between BMI and AF-related outcomes has not been reported. In view of the above-described inconsistencies, the complex interplay between HF and obesity and their frequent coexistence in patients with AF,^26^^,^^29^ we conducted the current study to assess whether the interaction between BMI and HF status (no HF, HFpEF, or HFrEF) has an impact on mortality, thromboembolism (ischemic stroke, systemic embolism, and transient ischemic attack [TIA]), myocardial infarction, and cardiovascular hospitalization, in a large, real-world cohort of patients with AF.

## Methods

### Study design and data sources

This cohort study was performed using data from the GWTG-AFIB (Get With The Guidelines-Atrial Fibrillation) registry, a national, voluntary quality improvement program initiated by the American Heart Association in June 2013, aiming to improve cardiovascular health and outcomes in patients with AF through adherence to guideline-recommended therapies.^30^ The GWTG-AFIB registry collects patient-level data from >150 participating hospitals across the United States, including sociodemographic and clinical characteristics, medical history, diagnosis, treatment, hospital characteristics, and in-hospital outcomes. We also used fee-for-service Medicare claims data from the U.S. Centers for Medicare & Medicaid Services (CMS) which include inpatient information on demographics, date of service, diagnoses recorded using the International Classification of Diseases (ICD)-9th Revision codes and the ICD-10th Revision codes (Supplemental Appendix), and date of death. Data from the GWTG-AFIB registry (2013-2023) and CMS (2013-2021) were linked through a procedure previously described.^31^

### Study cohort

We included patients admitted as an inpatient with a primary or secondary diagnosis of AF and discharged between January 1, 2013, and June 30, 2021, in 152 sites participating in the GWTG-AFIB registry. Our cohort was limited to patients aged ≥65 years who were linked to Medicare fee-for-service claims. We excluded patients with <6 months of calendar time for follow-up, those with missing information on HF status, BMI, left ventricular ejection fraction (LVEF), those who died during index admission, left against medical advice, or had missing or unknown discharge status. Patients entered our cohort at their first GWTG record meeting the study eligibility criteria.

### Main exposure variables

BMI (in kg/m^2^) was calculated as weight in kilograms divided by height in meters squared and classified as underweight (≤18.5), normal (18.5-24.9), overweight (25-29.9), and obesity (≥30). The acceptable range for BMI range was 7 to 150 kg/m^2^; any values outside of that range were excluded. HF was categorized according to LVEF as HFrEF (LVEF ≤40%), HFpEF (LVEF ≥50%), and HF with mid-range EF (HFmrEF, LVEF 41% to 49%), based on the most recent LVEF ACEi finding at/before discharge. To maintain enough statistical power and facilitate interpretation, patients with HFpEF and HFmrEF were analyzed in one group.

### Covariates

We abstracted information on demographic characteristics (ie, age, sex, race, and ethnicity); insurance status (categorized as private/health maintenance organization, Medicaid, Medicare, and Medicare-private health maintenance organization); type of AF (ie, first detected, paroxysmal, persistent, or permanent/long-standing persistent); medical history (ie, anemia, chronic obstructive pulmonary disease, coronary artery disease, diabetes mellitus, hypertension, left ventricular hypertrophy, sinus node dysfunction, rheumatic heart disease, mitral stenosis, peripheral vascular disease, stroke or TIA, hemorrhage, obstructive sleep apnea, liver disease, thyroid disease, hyperlipidemia, renal insufficiency, and smoking in the past 12 months); family history of AF; results of laboratory tests (ie, labile international normalized ratio and serum creatinine); previous procedures (percutaneous coronary intervention, pacemaker, implantable cardiac defibrillator, cardiac resynchronization therapy-D and -P, mechanical prosthetic heart valve), and discharge medications (ie, angiotensin-converting enzyme inhibitor, aldosterone antagonist, angiotensin II receptor blocker [ARB], angiotensin receptor-neprilysin inhibitor, β-blocker, calcium-channel blocker, antiarrhythmic, statin, antiplatelet, direct oral anticoagulant, and warfarin); hospital characteristics (ie, academic or teaching hospital, number of beds, patient volume discharges, rural location, and geographic region). Race and ethnicity were self-reported and classified in mutually exclusive categories as American Indian/Alaska Native, Native Hawaiian or Pacific Islander, Asian, Black, Hispanic (any race), and White.

### Outcomes

The primary outcomes were all-cause mortality within 30 days and 1 year of discharge. The secondary outcomes were cardiovascular rehospitalization (Supplemental Appendix), a composite of ischemic stroke, TIA, systemic embolism, and myocardial infarction, all within 30 days and 1 year of discharge. Follow-up for outcomes was censored early if the patient died, ended Medicare fee-for-service coverage, or at the end of CMS data availability (December 31, 2021).

### Statistical analysis

Summary statistics are reported as percentages for categorical variables and medians with 25th-75th percentiles (Q1-Q3) for continuous variables. Between-group differences were assessed using the chi-square test or Fisher exact test as appropriate for categorical variables and Kruskal-Wallis test for continuous variables. Absolute standardized differences were calculated as the absolute difference in means or proportions divided by a pooled estimate of the SD (and multiplied by 100 to get a percentage), with a percentage absolute standardized difference >10% indicating an imbalance between the study groups.

Death and adverse cardiovascular cumulative incidence rates were estimated with follow-up time starting on the date of index discharge and ending when the outcome occurred or at the censor date from CMS claims. We calculated the cumulative incidence for 30-day and 1-year postdischarge primary and secondary outcomes by BMI categories and stratified by HF status. Death cumulative incidence event rates were estimated using the Kaplan-Meier method and presented graphically. Nonfatal outcome rates were estimated using cumulative incidence functions that account for competing risk of death and are presented graphically. Differences by BMI categories within each stratum of HF status were assessed using the log-rank test for fatal endpoints and Gray’s test for nonfatal endpoints.

Cox proportional hazards models with robust variance estimation to account for within-hospital clustering were used to determine the association between BMI (as a continuous measure) and fatal outcomes, with adjustment for the following a priori specified potential confounders: demographics (age, sex, insurance status), medical history (CAD, prior stroke or TIA, diabetes, hemodialysis, hypertension, liver disease, obstructive sleep apnea, peripheral artery disease, chronic obstructive pulmonary disease, prior hemorrhage, prior myocardial infarction, prior percutaneous coronary intervention, smoker, thyroid disease), type of AF, admission estimated glomerular filtration rate, medications at discharge (beta-blocker, calcium-channel blocker, ACEi/ARB, ARNi, statin, antiarrhythmic, antiplatelet, direct oral anticoagulant, warfarin), and hospital characteristics (region, teaching/nonteaching, number of beds, rural/urban location). Cause-specific Cox proportional hazards regression models were used to account for the competing risk of death for nonfatal outcomes. Models were fit stratified by HF status group. First, we graphically assessed the functional form of BMI on each unadjusted time-to-event outcome using restricted cubic splines with 5 knots selected a priori^32^ and located at the 5th, 27.5th, 50th, 72.5th, and 95th percentiles of BMI distribution, and with BMI on the x-axis and log HR on the y-axis. The functional form was determined to be significantly nonlinear and, for simplicity of interpretation, was approximated with a linear spline (“broken stick model”) with a knot at BMI = 30 kg/m^2^, allowing us to characterize the HR for increments in BMI for values <30 and ≥ 30 kg/m^2^. Differences in association of BMI with outcomes by HFrEF vs no HF and HFpEF/HFmrEF vs no HF were assessed using interaction tests. We calculated HRs and 95% CIs from the adjusted models for comparisons of unit increments in BMI within each BMI category (BMI <30 and BMI ≥30). We performed a sensitivity analysis excluding patients with a BMI <18.5 or >50. The upper cutoff of BMI >50 was chosen because the 99th percentile of the overall distribution was 50.8. The handling of missing data for model covariates is described in the Appendix. All statistical tests were 2-tailed with 0.05 as the nominal significance level. All analyses were performed using SAS software (version 9.4, SAS Institute).

### Ethical considerations

This study was conducted under approval from the Institutional Review Board for Duke University, and under a waiver of informed consent.

## Results

### Population characteristics

From 36,500 patients in the GWTG-AF registry admitted for AF between January 1, 2013, and June 30, 2021, and with data linked to CMS, after applying exclusion criteria, 21,850 were finally included (Supplemental Table 1). Most patients did not have HF (68.6%), whereas 21.8% had HFpEF/HFmrEF and 9.6% had HFrEF. Selected patient baseline characteristics according to HF status are presented in Table 1, with more details presented in Supplemental Table 2. There were substantial differences in sociodemographic and clinical characteristics between patients without HF, with HFrEF, and HFpEF/HFmrEF. More than a third of patients were obese, and obesity was more commonly seen in patients with HFpEF/HFmrEF than those with HFrEF or without HF. Baseline characteristics by BMI categories are presented in Supplemental Table 3. Compared to patients underweight/normal weight (merged group), those overweight or obese were significantly younger, with lower CHA_2_DS_2_-VASc scores, and more commonly prescribed medications such as angiotensin-converting enzyme inhibitor, ARB, angiotensin receptor-neprilysin inhibitor, beta-blockers, statins, oral anticoagulant, and antiarrhythmic drugs.

**Table 1:** Patient Characteristics by Heart Failure Status

	No HF (n = 14,991)	HFrEF (n = 2,101)	HFpEF/HFmrEF (n = 4,758)	*P* Value	Standardized Difference Scores (%) (vs No HF)	
					HFrEF	HFpEF/HFmrEF
Age, y	77.0 (71.0-83.0)	77.0 (70.0-83.0)	79.0 (73.0-86.0)	<0.001	3.29	20.31
Male	6,582 (43.9)	1,299 (61.8)	1856 (39.0)	<0.001	36.49	9.96
Race and ethnicity						
Asian	155 (1.0)	28 (1.3)	62 (1.3)	0.19	2.76	2.50
Black	483 (3.2)	127 (6.0)	222 (4.7)	<0.001	13.46	7.42
Hispanic	523 (3.5)	85 (4.0)	145 (3.0)	0.099	2.93	2.48
NHPI	14 (0.1)	<11 (<0.5)	<11 (<0.2)	0.014	5.20	0.99
AIAN	29 (0.2)	12 (0.6)	13 (0.3)	0.004	6.12	1.65
White	13,921 (92.9)	1873 (89.1)	4,294 (90.2)	<0.001	13.01	9.41
Unable to determine	402 (2.7)	59 (2.8)	171 (3.6)	0.005	0.77	5.23
BMI (kg/m^2^)	27.8 (24.2-32.4)	27.6 (24.1-32.3)	28.9 (24.7-34.3)	<0.001	1.25	16.26
BMI categories						
Underweight	466 (3.1)	48 (2.3)	118 (2.5)	<0.001	5.90	15.91
Normal	4,063 (27.1)	601 (28.6)	1,156 (24.3)			
Overweight	4,935 (32.9)	693 (33.0)	1,362 (28.6)			
Obesity	5,527 (36.9)	759 (36.1)	2,122 (44.6)			
LVEF (%)	58.0 (52.0-63.0)	30.0 (23.0-35.0)	57.0 (50.0-62.0)	<0.001	239.64	7.50
Type of AF						
First detected AF	3,964 (26.4)	237 (11.3)	554 (11.6)	<0.001	51.07	45.10
Paroxysmal	6,282 (41.9)	794 (37.8)	2087 (43.9)			
Persistent	2,448 (16.3)	553 (26.3)	1,088 (22.9)			
Permanent	899 (6.0)	306 (14.6)	631 (13.3)			
Unable to determine	1,398 (9.3)	211 (10.0)	398 (8.4)			
Comorbidities						
Diabetes	3,874 (25.8)	736 (35.0)	1,620 (34.0)	<0.001	20.07	17.99
Hypertension	11,956 (79.8)	1,683 (80.1)	4,924 (84.6)	<0.001	0.87	12.61
Liver disease	123 (0.8)	26 (12)	69 (1.5)	0.004	4.13	5.95
Thyroid disease	3,020 (20.1)	414 (19.7)	1,208 (25.4)	<0.001	1.10	12.53
OSA	2089 (13.9)	366 (17.4)	1,005 (21.1)	<0.001	9.60	18.99
COPD	2,232 (14.9)	468 (22.3)	1,306 (27.4)	<0.001	19.08	31.12
Renal disease	806 (5.4)	246 (11.7)	525 (11.0)	<0.001	22.80	20.72
PAD	959 (6.4)	204 (9.7)	512 (10.8)	<0.001	12.20	15.63
CAD	4,092 (27.3)	1,107 (52.7)	2003 (42.1)	<0.001	53.67	31.48
Prior stroke or TIA	2,319 (15.5)	381 (18.1)	946 (19.9)	<0.001	7.13	11.59
Cigarette smoking	927 (6.2)	153 (7.3)	271 (5.7)	0.042	4.38	2.06
Discharge medications						
ACEI	3,510 (23.4)	726 (34.6)	986 (20.7)	<0.001	73.90	9.07
ARB	2,466 (16.4)	376 (17.9)	791 (16.6)	<0.001	59.13	6.23
ARNi	87 (0.7)	146 (8.0)	38 (0.9)	<0.001	54.86	4.33
Beta-blocker	9,779 (65.2)	1781 (84.8)	3,355 (70.5)	<0.001	63.18	13.60
CCA	5,013 (33.4)	244 (11.6)	1,657 (34.8)	<0.001	54.18	3.08
Statin	8,663 (57.8)	1,375 (65.4)	2,940 (61.8)	<0.001	21.47	13.29
Antiarrhythmic	6,245 (41.7)	918 (43.7)	1859 (39.1)	<0.001	6.69	6.00
Antiplatelet	6,510 (43.4)	1,104 (52.5)	2,198 (46.2)	<0.001	18.56	6.29
DOAC	9,264 (61.8)	1,173 (55.8)	2,561 (53.8)	<0.001	14.85	17.00
Warfarin	2,811 (18.8)	555 (26.4)	1,309 (27.5)	<0.001	18.53	22.39

ACEI = angiotensin-converting enzyme inhibitor; AF = atrial fibrillation; AIAN = American Indian or Alaska native; ARB = angiotensin II receptor blocker; ARNi = angiotensin receptor neprilysin inhibitor; BMI = body mass index; CAD = coronary artery disease; CCA = calcium-channel blocker; COPD = chronic obstructive pulmonary disease; DOAC = direct oral anticoagulant; HF = heart failure; HFmrEF = heart failure with mid-range ejection fraction; HFpEF = heart failure with preserved ejection fraction; HFrEF = heart failure with reduced ejection fraction; LVEF = left ventricular ejection fraction; NHPI = Native Hawaiian or Pacific Islander; OSA = obstructive sleep apnea; PAD = peripheral artery disease; TIA = transient ischemic attack.

Values are n (%) or median (25th-75th percentiles).

### Relationship of BMI with outcomes by HF status

#### Mortality

**Figure 1:**
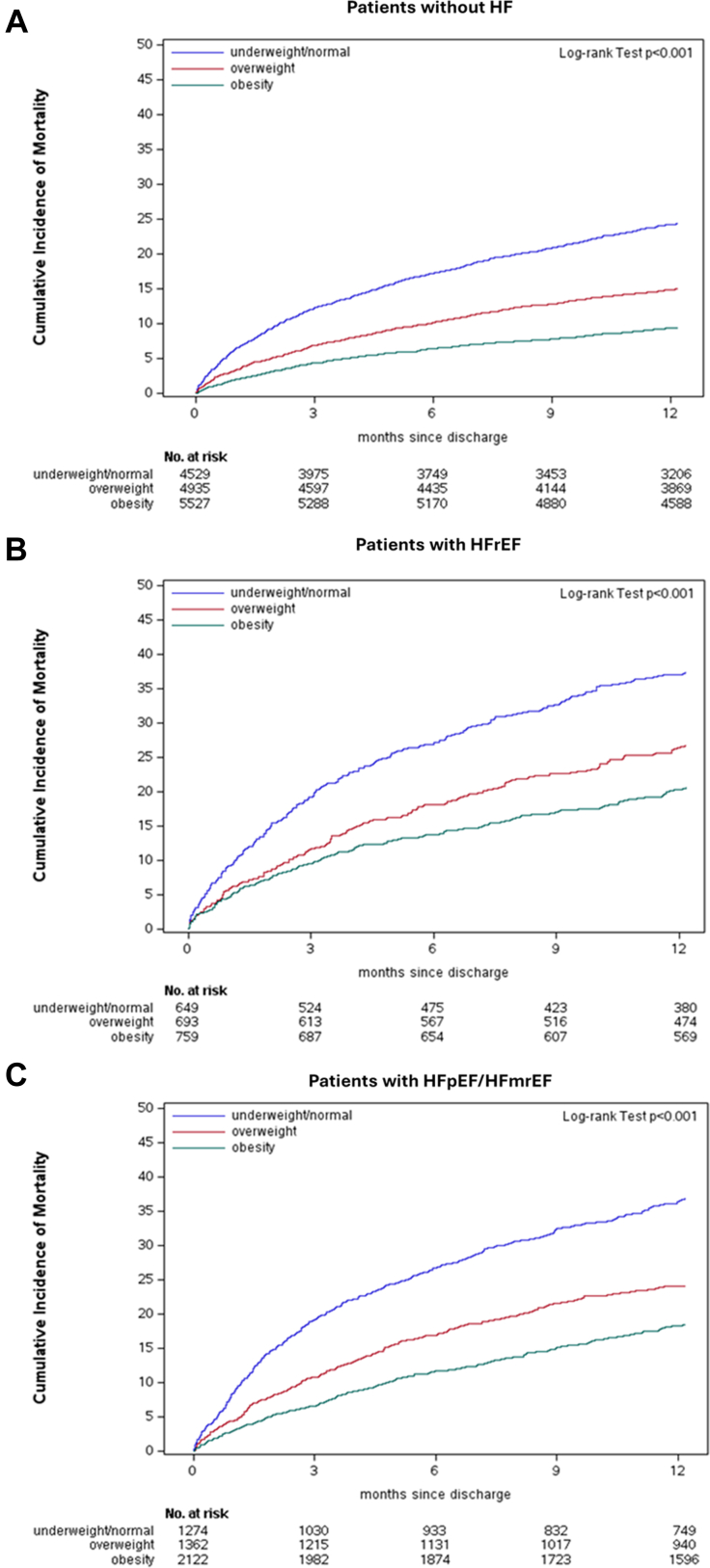
**Cumulative Incidence Curves for Death Across BMI Categories** (A) AF patients without HF; (B) patients with HFrEF; (C) patients with HFpEF/HFmrEF. AF = atrial fibrillation; BMI = body mass index; HF = heart failure; HFpEF = heart failure with preserved ejection fraction; HFmrEF = heart failure with mid-range ejection fraction; HFrEF = heart failure with reduced ejection fraction.

**Figure 2:**
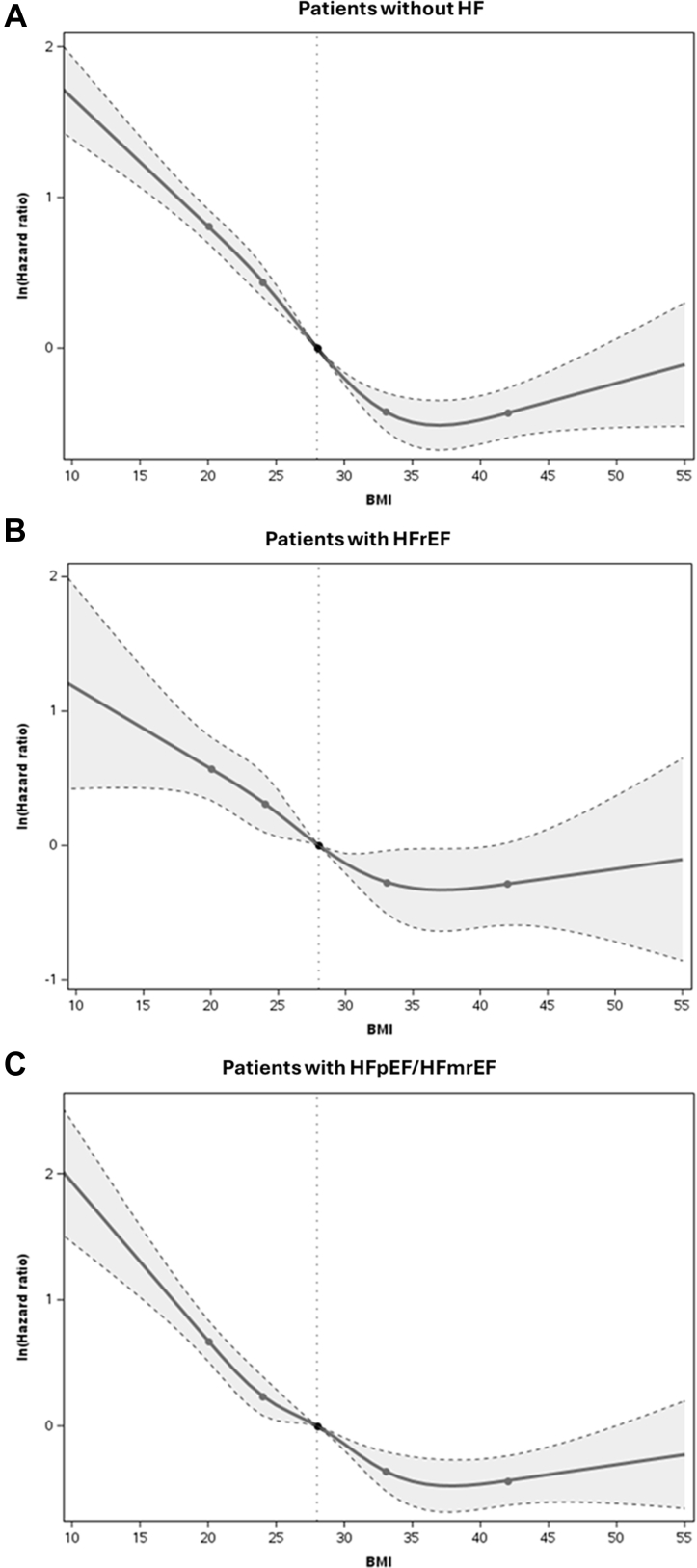
**Spline Plots Assessing the Unadjusted Association of BMI on Death Within 1 Year** (A) AF patients without HF; (B) patients with HFrEF; (C) patients with HFpEF/HFmrEF. Abbreviations as in Figure 1.

**Figure 3:**
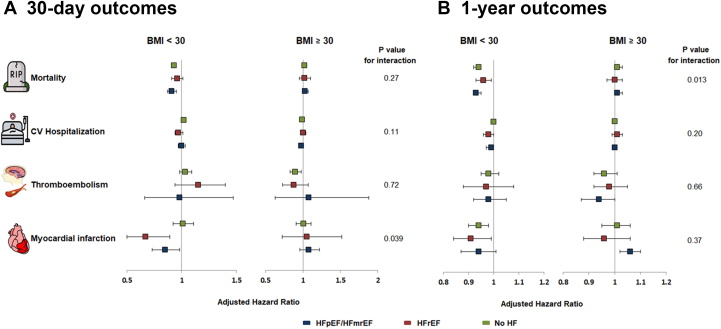
**Adjusted Association of BMI on Outcomes According to Heart Failure Status** (A) Corresponds to the two forest plots on the left side which represents 30-day outcomes. (B) Corresponds to the two forest plots on the right side which represents 1-year outcomes. Adjusted HR is per 1 kg/m^2^ increment in BMI. CV = cardiovascular; the other abbreviations as in Figure 1.

Unadjusted cumulative mortality incidence rates were consistently lower in obese compared with underweight/normal and overweight patients within the population without HF (*P* < 0.001), with HFrEF (*P* < 0.001), and with HFpEF/HFmrEF (*P* < 0.001) (Table 2, Figure 1). Spline plots showed lower risk of mortality within 1 year for increments in BMI up to 30 kg/m^2^, with no significant association of BMI with mortality above 30 kg/m^2^, and this was consistently observed in AF patients without HF (Figure 2A), with HFpEF/HFmrEF (Figure 2B) and HFrEF (Figure 2C). Similar findings were observed for mortality within 30 days (Supplemental Figure 1). In adjusted Cox regression analysis, across HF groups, increasing BMI was associated with lower 1-year mortality in patients without obesity, but there was little additional risk reduction for further increments in BMI among patients with obesity. Among patients without obesity, the risk reduction associated with BMI (per 1 kg/m^2^ increase) was slightly smaller in patients with HFrEF (HR: 0.96; 95% CI: 0.93-0.99) than in patients without HF (HR: 0.94; 95% CI: 0.92-0.95), and in patients with HFpEF/HFmrEF (HR: 0.93; 95% CI: 0.92-0.95) (interaction *P* = 0.013). The risk of mortality within 30 days was not significantly different across the HF spectrum between patients with and without obesity (interaction *P* = 0.27) (Figure 3, Supplemental Table 4).

**Table 2:** Cumulative Incidence of Clinical Outcomes by Body Mass Index Categories and Stratified by Heart Failure Status

Outcome	Time From Discharge	Effect	Cumulative Incidence (95% CI)		
			No HF	HFrEF	HFpEF/HFmrEF
Mortality	30 d	Underweight/normal	6.4 (5.7-7.1)	9.2 (7.0-11.5)	8.8 (7.2-10.3)
		Overweight	3.3 (2.8-3.8)	5.9 (4.2-7.7)	4.5 (3.4-5.6)
		Obesity	2.0 (1.7-2.4)	4.7 (3.2-6.3)	3.1 (2.4-3.8)
	1 y	Underweight/normal	24.4 (23.1-25.7)	37.4 (33.7-41.2)	36.8 (34.2-39.5)
		Overweight	15.0 (14.0-16.0)	26.7 (23.4-30.0)	24.1 (21.8-26.4)
		Obesity	9.4 (8.6-10.2)	20.6 (17.7-23.5)	18.4 (16.8-20.1)
Cardiovascular rehospitalization^a^	30 d	Underweight/normal	6.5 (5.8-7.3)	14.6 (11.9-17.5)	10.1 (8.5-11.9)
		Overweight	7.3 (6.6-8.1)	10.0 (7.9-12.5)	11.1 (9.5-12.9)
		Obesity	7.0 (6.3-7.7)	12.3 (10.0-14.9)	9.2 (8.0-10.5)
	1 y	Underweight/normal	23.8 (22.5-25.1)	39.2 (35.2-43.0)	33.5 (30.9-36.2)
		Overweight	24.4 (23.2-25.7)	39.3 (35.4-43.0)	32.9 (30.3-35.5)
		Obesity	24.6 (23.4-25.8)	38.9 (35.2-42.5)	35.4 (33.3-37.5)
Ischemic stroke, transient ischemic attack, and systemic embolism^a^	30 d	Underweight/normal	0.5 (0.3-0.7)	0.5 (0.1-1.3)	0.1 (0.0-0.4)
		Overweight	0.5 (0.3-0.7)	0.6 (0.2-1.5)	0.2 (0.1-0.6)
		Obesity	0.3 (0.2-0.5)	0.6 (0.2-1.4)	0.0 (0.0-0.3)
	1 y	Underweight/normal	2.4 (1.9-2.9)	2.5 (1.5-4.0)	2.3 (1.5-3.2)
		Overweight	2.1 (1.7-2.5)	2.3 (1.4-3.7)	1.6 (1.0-2.4)
		Obesity	1.4 (1.1-1.7)	2.2 (1.3-3.5)	1.7 (1.2-2.4)
Myocardial infarction^a^	30 d	Underweight/normal	0.1 (0.1-0.3)	1.1 (0.5-2.2)	0.4 (0.2-0.9)
		Overweight	0.3 (0.2-0.5)	0.2 (0.0-0.8)	0.3 (0.1-0.8)
		Obesity	0.2 (0.1-0.3)	0.1 (0.0-0.8)	0.1 (0.0-0.4)
	1 y	Underweight/normal	1.5 (1.1-1.9)	3.3 (2.1-5.0)	1.7 (1.1-2.6)
		Overweight	1.2 (0.9-1.5)	2.0 (1.1-3.4)	1.3 (0.8-2.1)
		Obesity	1.0 (0.8-1.3)	1.2 (0.6-2.2)	1.7 (1.2-2.4)

Abbreviations as in Table 1.

a Cumulative incidence was calculated accounting for death as a competing risk for these outcomes. For all other outcomes, cumulative incidence was calculated using Kaplan-Meier method.

#### Cardiovascular rehospitalization

**Figure 4:**
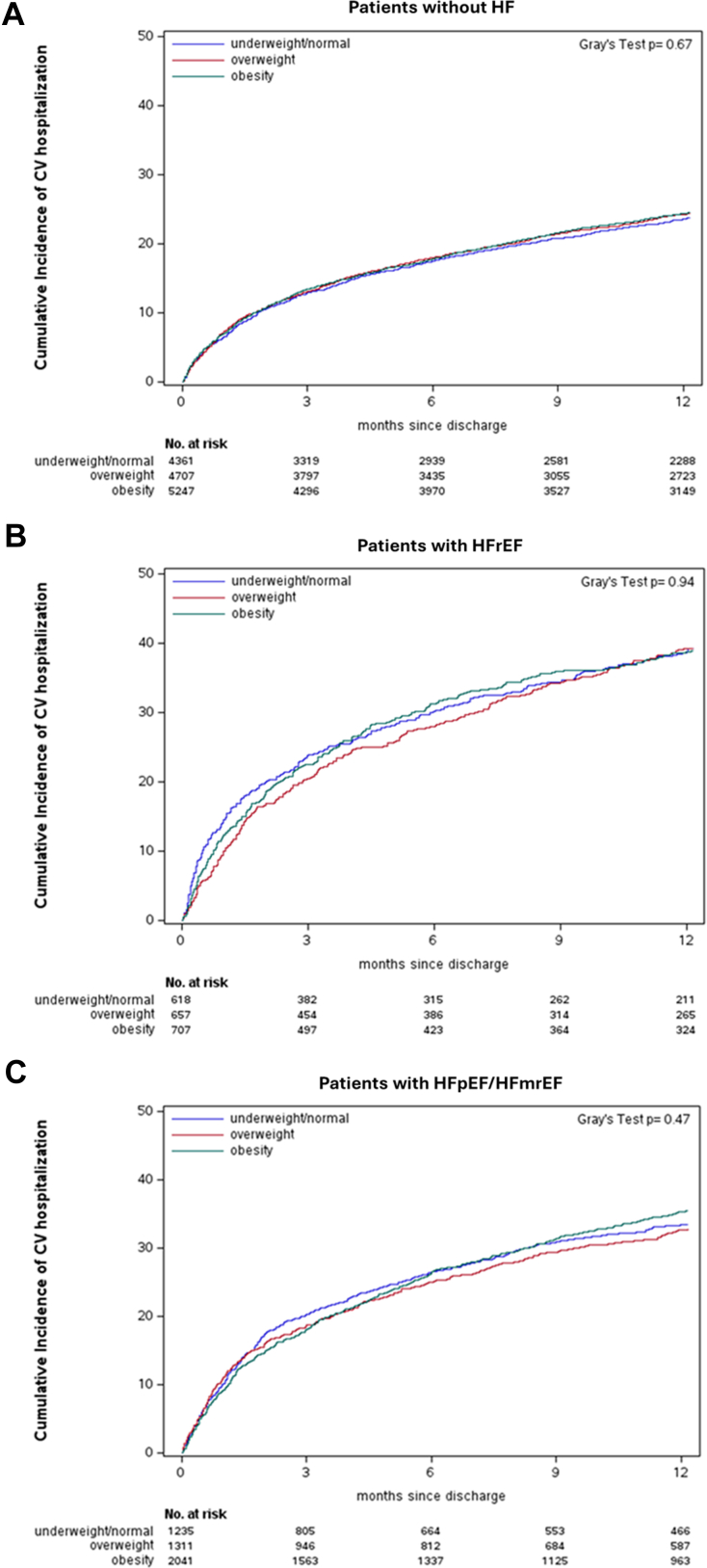
**Cumulative Incidence Curves for Cardiovascular Rehospitalization Across BMI Categories** (A) AF patients without HF; (B) patients with HFrEF; (C) patients with HFpEF/HFmrEF. Abbreviations as in Figure 1.

There was no difference in the cumulative incidence rates of cardiovascular rehospitalization between BMI categories in patients without HF, with HFrEF, and with HFpEF/HFmrEF (all *P* > 0.05) (Table 2, Figure 4). In adjusted Cox regression analysis, the risk of cardiovascular rehospitalization within 30 days and 1 year did not vary significantly between obese and nonpatients with obesity across the HF spectrum (all interaction *P* > 0.05) (Figure 3, Supplemental Table 4). Sensitivity analysis excluding underweight patients or with extremely high BMI (>50 kg/m^2^) showed similar results (Supplemental Table 5).

#### Ischemic stroke, TIA, and systemic embolism

The cumulative incidence rates of the composite of ischemic stroke, TIA, and systemic embolism were low across all BMI categories and HF status. Lower rates were observed in patients with obesity compared with underweight/normal and overweight patients within the population without HF (all *P* < 0.001) (Table 2, Supplemental Figure 2). The risks of ischemic stroke, TIA, and systemic embolism within 30 days and 1 year were not significantly different between patients with and without obesity across the HF spectrum (all interaction *P* > 0.05) in adjusted analysis in the full cohort (Figure 3, Supplemental Table 4), and after excluding underweight patients or with BMI >50 kg/m^2^ (Supplemental Table 5).

### Myocardial infarction

Unadjusted cumulative incidence rates of myocardial infarction were lower in obese compared with underweight/normal and overweight patients within the population with HFrEF (*P* = 0.027), with no difference across BMI categories in those without HF and with HFpEF/HFmrEF (all *P* > 0.05) (Table 2, Supplemental Figure 3). In adjusted Cox regression analysis, BMI (per 1 kg/m^2^ increase) was associated with lower 30-day risk of myocardial infarction in patients without obesity with HFpEF/HFmrEF (HR: 0.85; 95% CI: 0.73-0.98) and with HFrEF (HR: 0.67; 95% CI: 0.50-0.89) but not among patients with obesity (interaction *P* = 0.039). The risk of myocardial infarction within 1 year was not significantly different across the HF spectrum between patients with and without obesity (interaction *P* = 0.37) in the full cohort (Figure 3, Supplemental Table 4), and after excluding underweight patients or with BMI >50 kg/m^2^ (Supplemental Table 5).

## Discussion

In this cohort of patients with AF from a large national quality improvement registry, we observed that the crude mortality rates were consistently lower in patients with obesity compared to those underweight/normal weight and overweight irrespective of the HF status, whereas crude incidence rates of cardiovascular rehospitalization, thromboembolism (ischemic stroke, TIA, systemic embolism), and myocardial infarction were mostly not significantly different across BMI categories and HF status. After adjustment, increasing BMI was associated with lower 1-year mortality in patients without obesity, with or without HF, while increases in BMI among patients with obesity were not associated with lower mortality (Central Illustration). A lower risk of 30-day myocardial infarction with increasing BMI was observed in patients without obesity but with HF, with no significant association in patients with obesity.

**Central Illustration:**
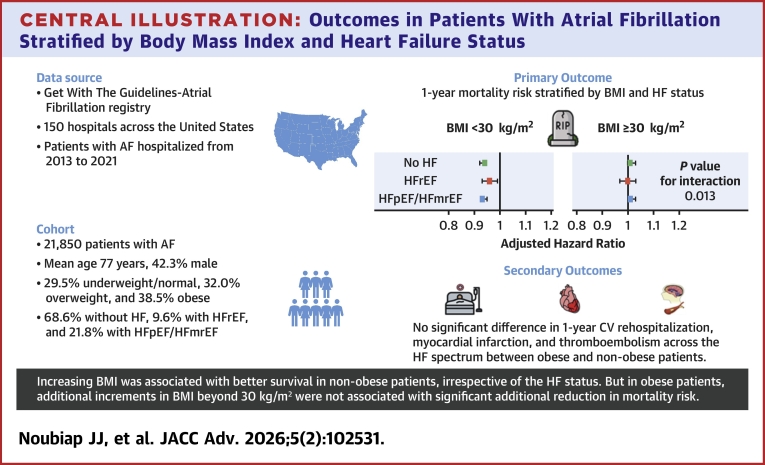
**Outcomes in Patients With Atrial Fibrillation Stratified by Body Mass Index and Heart Failure Status** Adjusted HR is per 1 kg/m^2^ increment in BMI. Abbreviations as in Figure 1.

We did not observe an obesity survival paradox. While this finding differs from observations from post hoc analyses of RCTs of oral anticoagulation which showed a lower risk of mortality associated with obesity,^24^, ^25^, ^26^, ^27^ it aligns with several reports from observational registries in which obesity was not associated with lower mortality.^27^, ^28^, ^29^ Although RCTs are considered the gold standard of clinical evidence for intervention effectiveness because their design minimizes bias and systematic error, they usually focus on relatively select populations that may not be representative of the general population. In contrast, observational registries cover broader populations without strict selection criteria and provide information that is closer to real-life clinical practice. Hence, real-word registries or cohorts are likely more suitable for prognostic studies.

A systematic review exploring the obesity paradox in HF showed that in most studies, patients with obesity were significantly younger than those with normal weight, had higher systolic blood pressure, higher LVEF, better renal function, less prevalent and less severe valvular regurgitation, better nutritional status, and more favorable values of other indicators of well-being.^33^ Similarly, in our study, patients with obesity were younger, had lower CHA_2_DS_2_-VASc scores, and were more commonly prescribed cardiovascular medications compared to their underweight/normal weight counterparts. This combination of favorable phenotypical traits and better treatment partly explains the higher survival observed in patients with obesity. Indeed, after adjusting for potential confounders, this apparent increased survival in patients with obesity was no longer observed in our study. It has been demonstrated that not accounting for some important but unidentified parameters can represent a statistical artifact and falsely suggest that obesity is associated with a more favorable prognosis.^34^

Although our findings are not consistent with an obesity survival paradox, we observed that increasing BMI was associated with lower mortality in patients with AF and a BMI <30, and this favorable effect was consistent in patients with and without HF. Higher BMI was also associated with lower risks of myocardial infarction and cardiovascular rehospitalization, but with variable effects depending on HF status. This is in keeping with previous evidence of increased thromboembolic and mortality risks in underweight patients with AF compared with those with normal weight.^27^ A potential explanation for these observations is that higher BMI in the nonobese population may be associated with greater metabolic reserve and increased ability to endure catabolic stress occurring with disease development and therefore, less adverse outcomes.^35^ Furthermore, underweight patients tend to be older, with higher comorbidity burden and frailty, factors that are associated with lower wellness and reduced survival.^36^

Our findings have important implications. The idea of an obesity survival paradox could cause clinicians to refrain from advising weight loss in patients with obesity. By showing that obesity is not independently associated with favorable outcomes, in the presence or absence of HF, our findings align with the current recommendations for weight loss in patients with obesity with AF.^19^ Indeed, it has been demonstrated that weight reduction in patients with obesity with AF reduces AF symptoms, reverses the type and natural progression of AF, and improves long-term freedom from AF.^20^, ^21^, ^22^, ^23^ Our findings also stress the importance of avoiding being underweight by maintaining a good nutritional status, as low BMI is associated with poorer outcomes.

### Study Limitations

Our study has some limitations. The use of administrative data and diagnostic codes is prone to ascertainment bias from coding errors, disease misclassification, and incomplete documentation. However, prior research has shown a high degree of accuracy of ICD codes in identifying conditions explored in this study.^37^, ^38^, ^39^, ^40^, ^41^ As with all observational studies, although we adjusted for known prognostic factors, we cannot exclude residual or unmeasured confounding, reverse causality, and collider bias, limiting confident conclusions regarding causal inference. For instance, frailty, functional status, socioeconomic status, and physical activity levels are not measured. BMI may partly act as a proxy for these unmeasured confounders, particularly in elderly populations. Furthermore, our findings might not be fully generalizable due to limited age, racial, and ethnic representativeness, as most of our study population was older and White, and due to potential selection bias, as the GWTG-AFIB program attracts hospitals interested in quality improvement. Because of the low event rates for certain outcomes, some analyses may have been underpowered, as reflected by the wide CIs, which limit the strength of the corresponding conclusions. To ensure adequate statistical power to detect differences between groups and for ease of interpretation, we merged patients with HFpEF and those with HFmrEF into one category. Therefore, the influence of these individual HF phenotypes on the relationship between BMI and AF-related outcomes was not explored. Moreover, because <3% of our study population was underweight and could not be analyzed as a separate group due to limited power, our findings cannot be extrapolated specifically to underweight patients. It is also worth noting that BMI was measured only at baseline, and therefore the impact of changes in adiposity over time on outcomes could not be evaluated. However, prior work on adiposity has shown that BMI trajectories remain fairly stable over time.^42^ Furthermore, BMI does not account for the location of fat or its amount relative to muscle or the weight of the skeleton and could also be falsely raised due to fluid retention in patients with HF. In fact, alternative anthropometric measurements such as waist-to-height ratio better reflect the location and amount of ectopic fat compared with BMI, and no evidence for an obesity-survival benefit was observed when using the waist-to-height ratio in patients with HFrEF.^9^ Despite these drawbacks, BMI is the more commonly used measure of adiposity in clinical practice, and alternative indices such as the waist circumference, waist-to-hip ratio, or weight-adjusted-weight index are more complex and are associated with high measurement error and interobserver variability.^43^

## Conclusions

Although patients with obesity exhibited lower crude mortality rates compared with patients without obesity, after adjustment, increasing BMI was associated with better survival in patients without obesity, irrespective of the HF status. But in patients with obesity, additional increments in BMI beyond 30 kg/m^2^ were not associated with significant additional reduction in mortality risk. This reinforces the recommendations to maintain a normal weight in these patients.

Perspectives **COMPETENCY IN MEDICAL KNOWLEDGE:** Although several studies showed an obesity-survival benefit, called the “obesity paradox” in patients with HF and those with AF, in this real-world cohort, we observed that increasing BMI was associated with better survival in patients without obesity, irrespective of the HF status, but not in patients with obesity.

**TRANSLATIONAL OUTLOOK:** This does not support the existence of an obesity-survival benefit in patients with AF but rather reinforces the recommendations to maintain a normal weight in these patients.

## Funding support and author disclosures

The GWTG-AFib program is provided by the American Heart Association. GWTG-AFib is sponsored, in part, by Novartis and BMS Pfizer. The funders had no role in the current study. Dr Jackson reports receiving consulting fees from Sanofi, Biosense Webster Inc, Johnson & Johnson Inc, Bristol Myers Squibb, and PRIME Education. Dr Russo reports receiving consulting fees from Abbott, Bayer, Biosense Webster, Biotronik, BMS-Pfizer, Boston Scientific, Medtronic, and Sanofi; research support from Abbott, Bayer, Boston Scientific, Medtronic, and Novartis. Dr Turchioe reports equity/co-founder status with Iris OB Health. Dr Volgman reports stock in Apple Inc; receiving consulting fees from Sanofi and Zoll; research support from Novartis and Jannsen. Dr Sanders reports having served on the advisory board of Medtronic, Abbott Medical, Boston Scientific, CathRx, and PaceMate; that the Adelaide University has received on his behalf research funding, lecture, and consulting fees from 10.13039/100004374Medtronic, 10.13039/100011953Abbott Medical, 10.13039/100008497Boston Scientific, and 10.13039/501100018918Microport. All other authors have reported that they have no relationships relevant to the contents of this paper to disclose.
