# Supplementary material for: Outcomes in Patients With Atrial Fibrillation Stratified by Body Mass Index and Heart Failure Status
Source: JACC Adv. 2026 Jan 19;5(2):102531. doi: 10.1016/j.jacadv.2025.102531 (PMC12856322; doi:10.1016/j.jacadv.2025.102531)
Supplement: Supplementary Material [file mmc1.pdf]

APPENDIX

Outcomes in Patients with Atrial Fibrillation stratified by Body Mass Index and Heart Failure Status

I. Notes on Diagnosis and Procedure Codes

A. ICD-9 and ICD-10 codes used to define conditions

Notes

Sources. This document contains definitions of conditions, using ICD-9-CM and ICD-10-CM diagnosis and procedure codes, that have been used in analyses for several registries linked to CMS data.

Codes for a number of conditions are given in the Quan paper; others are defined by the Chronic Conditions Warehouse. Definitions taken from one of these sources are noted as:

[QC] Quan paper, ICD-10 and enhanced ICD-9-CM columns of Table 1 (Charlson Comorbidities)

[QE] Quan paper, ICD-10 and enhanced ICD-9-CM columns of Table 2 (Elixhauser Comorbidities)

Quan H et al. Coding algorithms for defining comorbidities in ICD-9-CM and ICD-10 administrative data. Med Care. 2005; 43:1130-1139.

[CCW] Chronic Conditions Warehouse (ccwdata.org)

Diagnosis and procedure codes

- ICD-9-CM diagnosis codes have 3 digits to the left of the decimal, e.g. 410.01.
- ICD-9-CM procedure codes have 2 digits to the left of the decimal, e.g., 36.2
- ICD-10-CM diagnosis codes begin with a letter, e.g. Z86.74.
- ICD-10-PCS procedure codes begin with a number, e.g., 5A12012.

US vs. international

- The US system (with the CM suffix) can be found at icd9data.com and icd10data.com.
- US CM codes are not always the same as those in the original ICD-9 and ICD-10 used internationally. International ICD-10 codes can be found at https://icd.who.int/browse10/2019/en. ICD-9 codes are no longer available online.

Defining Outcomes

Patient is considered to have met the endpoint if one of the ICD-9-CM or ICD-10-CM codes occurs the primary position (first two positions for bleeding), at any time after the index date [or index discharge].

Conditions that are likely to result in hospitalization (e.g., MI, stroke) are defined solely from inpatient claims.

B. Cardiovascular conditions

Conditions	ICD-9-CM diagnosis and procedure codes	ICD-10-CM diagnosis and ICD-10-PCS procedure codes
**Cardiac conditions**		
Myocardial infarction	410.x1 Acute myocardial infarction, initial episode of care	I21.x Acute myocardial infarction
Congestive heart failure<br><br>[QC/QE]	398.91 Rheumatic heart failure (congestive)<br>402.01 Malignant hypertensive heart disease with heart failure<br>402.11 Benign hypertensive heart disease with heart failure<br>402.91 Unspecified hypertensive heart disease with heart failure<br>404.01, 404.11, 404.91 Hypertensive heart and chronic kidney disease, with heart failure and with chronic kidney disease stage I through stage IV, or unspecified<br>404.03, 404.13, 404.93 Hypertensive heart and chronic kidney disease, with heart failure and with chronic kidney disease stage V or end stage renal disease<br>425.2 Obscure cardiomyopathy of Africa<br>425.4 Other primary cardiomyopathies<br>425.5 Alcoholic cardiomyopathy<br>425.7 Nutritional and metabolic cardiomyopathy<br>425.9 Secondary cardiomyopathy, unspecified<br>428.xx Heart failure	I09.81 Rheumatic heart failure<br>I11.0 Hypertensive heart disease with heart failure<br>I13.0 Hypertensive heart and chronic kidney disease with heart failure and stage 1 through stage 4 chronic kidney disease, or unspecified chronic kidney disease<br>I13.2 Hypertensive heart and chronic kidney disease with heart failure and with stage 5 chronic kidney disease, or end stage renal disease<br>I42.0 Dilated cardiomyopathy<br>I42.5 Other restrictive cardiomyopathy<br>I42.6 Alcoholic cardiomyopathy<br>I42.7 Cardiomyopathy due to drug and external agent<br>I42.8 Other cardiomyopathies<br>I42.9 Cardiomyopathy, unspecified<br>I43 Cardiomyopathy in diseases classified elsewhere<br>I50.1 Left ventricular failure, unspecified<br>I50.2x Systolic (congestive) heart failure<br>I50.3x Diastolic (congestive) heart failure<br>I50.4x Combined systolic (congestive) and diastolic (congestive) heart failure<br>I50.9 Heart failure, unspecified
**Cardiac procedures**		
Percutaneous coronary intervention	00.66 PTCA<br>36.0x Removal of coronary artery obstruction and insertion of stent(s)	For n=0, 1, 2, 3<br>027nxxx Dilation of coronary arteries with/without drug-eluting/non-drug-eluting coronary stent<br>02Cnxxx Extirpation of matter from coronary arteries<br>3E07017, 3E070PZ, 3E07317, 3E073PZ Introduction of platelet inhibitor or other thrombolytic into coronary artery
Coronary artery bypass graft	36.1x Bypass anastomosis for heart revascularization<br>36.2 Heart revascularization by arterial implant<br>36.3x Transmyocardial revascularization; other revascularization	For n=0, 1, 2, 3<br>021nxxx Bypass of coronary arteries<br>021Kxxx Bypass of right ventricle

Conditions	ICD-9-CM diagnosis and procedure codes	ICD-10-CM diagnosis and ICD-10-PCS procedure codes
		021Lxxx Bypass of left ventricle<br>02QA0ZZ, 02QB0ZZ, 02QC0ZZ Repair heart, open approach
**Stroke**		
Stroke	430 Subarachnoid hemorrhage<br>431 Intracerebral hemorrhage<br>432.x Other and unspecified intracranial hemorrhage<br>433.x1 Occlusion and stenosis of precerebral arteries, with cerebral infarction<br>434.x1 Occlusion of cerebral arteries, with cerebral infarction<br>436 Acute but ill-defined cerebrovascular disease<br>437.1 Other generalized ischemic cerebrovascular disease<br>997.02 Iatrogenic cerebrovascular infarction or hemorrhage	I60.x Nontraumatic subarachnoid hemorrhage<br>I61.x Nontraumatic intracerebral hemorrhage<br>I62.xx Other and unspecified nontraumatic intracranial hemorrhage<br>I63.xxx Cerebral infarction<br>I67.81 Acute cerebrovascular insufficiency<br>I67.82 Cerebral ischemia<br>I67.89 Other cerebrovascular disease<br>I97.811 Intraoperative cerebrovascular infarction during other surgery<br>I97.821 Postprocedural cerebrovascular infarction following other surgery
Ischemic stroke	433.x1 Occlusion and stenosis of precerebral arteries, with cerebral infarction<br>434.x1 Occlusion of cerebral arteries, with cerebral infarction<br>436 Acute but ill-defined cerebrovascular disease	I63.xxx Cerebral infarction<br>I67.89 Other cerebrovascular disease
Hemorrhagic stroke	430 Subarachnoid hemorrhage<br>431 Intracerebral hemorrhage	I60.x Nontraumatic subarachnoid hemorrhage<br>I61.x Nontraumatic intracerebral hemorrhage
Transient ischemic attack	435.x Transient cerebral ischemia	G45.0 Vertebro-basilar artery syndrome<br>G45.1 Carotid artery syndrome (hemispheric)<br>G45.2 Multiple and bilateral precerebral artery syndromes<br>G45.8 Other transient cerebral ischemic attacks and related syndromes<br>G45.9 Transient cerebral ischemic attack, unspecified
Cerebrovascular disease<br><br>[QC]	362.34 Transient retinal arterial occlusion<br>430 Subarachnoid hemorrhage<br>431 Intracerebral hemorrhage<br>432.x Other and unspecified intracranial hemorrhage<br>433.xx Occlusion and stenosis of precerebral arteries<br>434.xx Occlusion of cerebral arteries<br>435.x Transient cerebral ischemia<br>436 Acute but ill-defined cerebrovascular disease<br>437.x Other and ill-defined cerebrovascular disease<br>438.xx Late effects of cerebrovascular disease	G45.x Transient cerebral ischemic attacks and related syndromes<br>G46.x Vascular syndromes of brain in cerebrovascular diseases<br>H34.0x Transient retinal artery occlusion<br>I60.xx Nontraumatic subarachnoid hemorrhage<br>I61.x Nontraumatic intracerebral hemorrhage<br>I62.xx Other and unspecified nontraumatic intracranial hemorrhage<br>I63.xxx Cerebral infarction<br>I65.xx Occlusion and stenosis of precerebral arteries, not resulting in cerebral infarction<br>I66.xx Occlusion and stenosis of cerebral arteries, not resulting in cerebral infarction

Conditions	ICD-9-CM diagnosis and procedure codes	ICD-10-CM diagnosis and ICD-10-PCS procedure codes
		I67.xxx Other cerebrovascular diseases<br>I68.x Cerebrovascular disorders in diseases classified elsewhere<br>I69.xxx Sequelae of cerebrovascular disease
**Vascular conditions**		
Systemic embolism	444.xx Arterial embolism and thrombosis	I74.xx Arterial embolism and thrombosis
Peripheral arterial disease	440.xx Atherosclerosis<br>441.xx Aortic aneurysm and dissection<br>442.xx Other aneurysm<br>443.x Other peripheral vascular disease<br>444.xx Arterial embolism and thrombosis<br>445.xx Atheroembolism<br>449 Septic arterial embolism<br>557.1 Chronic vascular insufficiency of intestine<br>996.74 Other complications due to other vascular device, implant and graft<br>997.2 Peripheral vascular complications, not elsewhere classified<br>997.7x Vascular complications of other vessels	I70.xxx Atherosclerosis<br>I71.xx Aortic aneurysm and dissection<br>I72.xx Other aneurysm<br>I73.x Other peripheral vascular diseases<br>I74.xx Arterial embolism and thrombosis<br>I75.xx Atheroembolism<br>I76 Septic arterial embolism<br>K55.1 Chronic vascular disorders of intestine<br>T82.3xxxMechanical complication of other vascular grafts<br>T82.5xxx Mechanical complication of other cardiac and vascular devices and implants<br>T82.8xxx Other specified complications of cardiac and vascular prosthetic devices, implants and grafts<br>T82.9xxx Unspecified complications of cardiac and vascular prosthetic devices, implants and grafts<br>T81.7xxxVascular complications following a procedure, not elsewhere classified

C. Cardiovascular readmission

Patient is considered to have a cardiovascular readmission if patient has

- MI, heart failure, stroke, ICH, TIA, cerebrovascular disease, PAD, PCI, or CABG as defined above **OR**
- any of the ICD-9 or ICD-10 codes below occurs in the primary diagnosis (or procedure) position in an acute care inpatient claim after the discharge date from the index hospitalization.

Notes

- Certain definitions (cardiac arrhythmia, operations) use procedure codes in addition to or instead of diagnosis codes.
- *Rows can break across pages, so check the next page to see the complete list.*

**Readmission reason**	**ICD-9-CM diagnosis or procedure codes**	**ICD-10-CM diagnosis or ICD-10-PCS procedure codes**
Unstable angina	411.1 Intermediate coronary syndrome	I20.0 Unstable angina
Chest pain	413.x Angina pectoris<br>786.5x Chest pain	I20.1, I20.8, I20.9 Angina pectoris (other than unstable)<br>R07.1, R07.2, R07.8x, R07.9 Chest pain
Prior MI or CAD	412 Old myocardial infarction<br>414.xx Other forms of chronic ischemic heart disease<br>429.7x Certain sequelae of myocardial infarction not elsewhere classified	I22.x Subsequent ST elevation (STEMI) and non-ST elevation (NSTEMI) myocardial infarction<br>I23.x Certain current complications following ST elevation (STEMI) and non-ST elevation (NSTEMI) myocardial infarction (within the 28 day period)<br>I25.xxx Chronic ischemic heart disease<br>(includes I25.2 Old myocardial infarction)<br>I51.0 Cardiac septal defect, acquired
Cardiac arrhythmia	426.xx Conduction disorders<br>427.xx Cardiac dysrhythmias<br>785.0 (Symptoms) Tachycardia, unspecified<br>785.1 (Symptoms) Palpitations<br>99.61 Atrial cardioversion<br>99.62 Other electric countershock of heart<br>99.69 Other conversion of cardiac rhythm	I44.xx Atrioventricular and left bundle-branch block<br>I45.xx Other conduction disorders<br>I46.x Cardiac arrest<br>I47.x Paroxysmal tachycardia<br>I48.xx Atrial fibrillation and flutter<br>I49.xx Other cardiac arrhythmias<br>R00.2 Palpitations<br>5A2204Z Restoration of cardiac rhythm, single
Valvular disease	391.1 Acute rheumatic endocarditis<br>394.x Diseases of mitral valve	I01.1 Acute rheumatic endocarditis<br>I05.x Rheumatic mitral valve diseases

Readmission reason	ICD-9-CM diagnosis or procedure codes	ICD-10-CM diagnosis or ICD-10-PCS procedure codes
	395.x Diseases of aortic valve<br>396.x Disease of mitral and aortic valves<br>397.x Diseases of other endocardial structures<br>398.9x Other and unspecified rheumatic heart diseases<br>421.x Acute and subacute endocarditis<br>424.xx Other diseases of endocardium<br>429.5 Rupture of chordae tendinae<br>429.6 Rupture of papillary muscle<br>785.2 Undiagnosed cardiac murmurs	I06.x Rheumatic aortic valve diseases<br>I07.x Rheumatic tricuspid valve diseases<br>I08.x Multiple valve diseases<br>I09.1 Rheumatic diseases of endocardium, valve unspecified<br>I09.8x, I09.9 Other and unspecified rheumatic heart diseases<br>I33.x Acute and subacute endocarditis<br>I34.x Nonrheumatic mitral valve disorders<br>I35.x Nonrheumatic aortic valve disorders<br>I36.x Nonrheumatic tricuspid valve disorders<br>I37.x Nonrheumatic pulmonary valve disorders<br>I38 Endocarditis, valve unspecified<br>I39 Endocarditis and heart valve disorders in diseases classified elsewhere<br>I51.1 Rupture of chordae tendinae, not elsewhere classified<br>I51.2 Rupture of papillary muscle, not elsewhere classified<br>R01.1 Cardiac murmur, unspecified
Hypertensive heart disease	401.x Essential hypertension<br>402.xx Hypertensive heart disease<br>403.xx Hypertensive chronic kidney disease<br>404.xx Hypertensive heart and chronic kidney disease<br>405.xx Secondary hypertension	I10 Essential (primary) hypertension<br>I11.x Hypertensive heart disease<br>I12.x Hypertensive chronic kidney disease<br>I13.xx Hypertensive heart and chronic kidney disease<br>I15.x Secondary hypertension
Myocarditis or pericarditis	391.0 Acute rheumatic pericarditis<br>391.2 Acute rheumatic myocarditis<br>391.8 Other acute rheumatic heart disease<br>391.9 Acute rheumatic heart disease, unspecified<br>398.x Other rheumatic heart disease<br>411.0 Postmyocardial infarction syndrome<br>414.1x Aneurysm and dissection of heart<br>420.xx Acute pericarditis<br>422.xx Acute myocarditis<br>423.x Other diseases of pericardium<br>429.0 Myocarditis, unspecified	I01.0 Acute rheumatic pericarditis<br>I01.2 Acute rheumatic myocarditis<br>I01.8 Other acute rheumatic heart disease<br>I01.9 Acute rheumatic heart disease, unspecified<br>I09.0 Rheumatic myocarditis<br>I09.2 Chronic rheumatic pericarditis<br>I09.8x, I09.9 Other and unspecified rheumatic heart diseases<br>I24.1 Dressler's syndrome<br>I25.3 Aneurysm of heart<br>I25.4x Coronary artery aneurysm and dissection<br>I30.x Acute pericarditis<br>I31.x Other diseases of pericardium<br>I32 Pericarditis in diseases classified elsewhere

Readmission reason	ICD-9-CM diagnosis or procedure codes	ICD-10-CM diagnosis or ICD-10-PCS procedure codes
		I40.x Acute myocarditis<br>I41 Myocarditis in diseases classified elsewhere
Cardiovascular disease unspecified	411.8x Other acute and subacute forms of ischemic heart disease<br>429.1 Myocardial degeneration<br>429.2 Cardiovascular disease, unspecified<br>429.3 Cardiomegaly<br>429.4 Functional disturbances following cardiac surgery<br>429.8x Other ill-defined heart diseases<br>429.9 Heart disease, unspecified<br>785.0 Tachycardia, unspecified<br>785.1 Palpitations<br>785.2 Undiagnosed cardiac murmurs<br>785.3 Other abnormal heart sounds<br>785.5x Shock without mention of trauma<br>785.9 Other symptoms involving cardiovascular system	I24.x Other acute ischemic heart diseases<br>I25.9 Chronic ischemic heart disease, unspecified<br>I51.xx Complications and ill-defined descriptions of heart disease (includes Myocardial degeneration [I51.5], Cardiomegaly [I51.7], and Heart disease unspecified [I51.9])<br>I97.0 Postcardiotomy syndrome<br>I97.1xx Other postprocedural cardiac functional disturbances<br>R00.2 Palpitations<br>R01.1 Cardiac murmur, unspecified<br>R01.2 Other cardiac sounds<br>R57.x Shock, not elsewhere classified (not trauma)<br>R09.89 Other specified symptoms and signs involving the circulatory and respiratory systems
Procedural cardiac complications	996.0x Mechanical complication of cardiac device implant and graft<br>996.1 Mechanical complication of other vascular device, implant, or graft<br>997.1 Cardiac complications, not elsewhere classified<br>997.2 Peripheral vascular complications, not elsewhere classified	I97.xxx Intraoperative and postprocedural complications and disorders of circulatory system, not elsewhere classified<br>T81.7xxx Vascular complications following a procedure, not elsewhere classified<br>T82.xxxx Complications of cardiac and vascular prosthetic devices, implants and grafts
Operations on cardiac valves and septa	35.xx Operations on valves and septa of heart (includes structures adjacent to valves)	For n=F, G, H, J, 5, 9, D, M<br>02xnxxx Operations on valves, adjacent structures, or septa
Operations on coronary vessels	36.xx Operations on vessels of heart	For n=0, 1, 2, 3, 4<br>02xnxxx Operations on coronary arteries or coronary vein
Operations on heart or pericardium	37.xx Other operations on heart and pericardium	For n=A, 6, 7, 8, K, L, N<br>02xnxxx Operations on heart, atria, ventricles, conduction mechanism, or pericardium<br>0W9Cxxx Drainage of mediastinum<br>0W9Dxxx Drainage of pericardial cavity<br>3E08xxx Introduction of therapeutic substance into heart<br>4A02xxx Cardiac measurement (including cath)<br>5A02xxx Assistance with cardiac output using pump

Readmission reason	ICD-9-CM diagnosis or procedure codes		ICD-10-CM diagnosis or ICD-10-PCS procedure codes	
Operations — other cardiovascular	17.5x	Additional cardiovascular procedures	02HKxxx	Insertion of device into right ventricle
	38.x	Incision, excision, and occlusion of vessels	03xxxxx	Procedure on an upper (non-coronary) artery
	39.x	Other operations on vessels	04xxxxx	Procedure on a lower artery
	99.6x	Conversion of cardiac rhythm	05xxxxx	Procedure on an upper vein
			06xxxxx	Procedure on a lower vein
			3E03xxx	Introduction of therapeutic substance into peripheral vein
			3E04xxx	Introduction of therapeutic substance into central artery
			5A12xxx	Cardiopulmonary resuscitation
			5A22xxx	Restoration of cardiac rhythm

II. Handling of Missing Data for model covariates:

We tabulated the percentage missing each covariate as well as the percentage of patients missing at least one covariate. For missing medical history variables, we assumed the condition did not occur. Missing hospital characteristics were imputed using single imputation with missing urban/rural imputed to urban (the most frequent category), academic/teaching imputed to no (assuming non-academic sites may ignore the academic/teaching status check box), and missing bed size was imputed to 430 for academic sites and 210 for non-academic sites. Patients at sites with missing region were excluded from models. The percentage of patients missing at least one of the remaining covariates was ≤5%, and we used single imputation to fill in missing values. For eGFR, missing values and values greater than the 99th percentile were imputed to the population median. For BMI, values greater than the 99th percentile were imputed to the 99th percentile.

III. Results

Table S1. Patient selection

Inclusion/Exclusion	Remaining<br>N Patients Remaining	 N Sites	Excluded<br>N Patients Excluded	 N Sites Excluded	 % Patients Excluded
1) Starting Data: GWTG-AFIB pts linked to CMS and admitted as inpatient for AFIB	36,500	152	(.)	(.)	.
2) Include pts w/ at least 6 months of calendar time for f/u* (Index discharge <=30June2021)	34,477	151	(2,023)	(1)	5.54
3) Exclude missing history of HF status	34,410	151	(67)	(0)	0.19
4) Exclude missing BMI	28,448	133	(5,962)	(18)	17.33
5) Exclude missing LVEF	24,915	133	(3,533)	(0)	12.42
6) Exclude pts who died during index visit, left AMA, or have missing/unknown discharge status	24,452	133	(463)	(0)	1.86
7) First record per patient	21,850	133	(2,602)	(0)	10.64

*Follow-up for outcomes was collected post-discharge through 31DEC2021 or administrative censor date.

Table S2. Patient characteristics by heart failure status

Variable	Level	No HF (N=14991)		HFrEF (N=2101)		HFpEF/HFmrEF (N=4758)		P-value+	Std Dif Score* (versus No HF) HFrEF	Std Dif Score* (versus No HF) HFpEF/HFmrEF
**Demographics**										
Age++ /ranks	Median	14991	77.0	2101	77.0	4758	79.0	<0.001	3.29	20.31
	25th		71.0		70.0		73.0			
	75th		83.0		83.0		86.0			
	Mean		77.5		77.3		79.2			
	STD		8.0		8.1		8.3			
	Missing(%)		0.0		0.0		0.0			
Age++ /ranks	<75	6041	40.3	869	41.4	1546	32.5	<0.001	2.16	16.28
	>=75	8950	59.7	1232	58.6	3212	67.5			
Sex	Male	6582	43.9	1299	61.8	1856	39.0	<0.001	36.49	9.96
	Female	8409	56.1	802	38.2	2902	61.0			
BMI (kg/m^2^)++ /ranks	Median	14991	27.8	2101	27.6	4758	28.9	<0.001	1.25	16.26
	25th		24.2		24.1		24.7			
	75th		32.4		32.3		34.3			
	Mean		28.9		28.8		30.3			
	STD		7.8		6.9		9.0			
	Missing(%)		0.0		0.0		0.0			
BMI categories	underweight	466	3.1	48	2.3	118	2.5	<0.001	5.90	15.91
	normal	4063	27.1	601	28.6	1156	24.3			
	overweight	4935	32.9	693	33.0	1362	28.6			
	obesity	5527	36.9	759	36.1	2122	44.6			

Variable	Level	No HF		HFrEF		HFpEF/HFmrEF		P-value+	Std Dif Score* (versus No HF)	
		(N=14991)		(N=2101)		(N=4758)			HFrEF	HFpEF/HFmrEF
Race^: American Indian or Alaska Native	No	14962	99.8	2089	99.4	4745	99.7	0.004	6.12	1.65
	Yes	29	0.2	12	0.6	13	0.3			
Race^: Asian	No	14836	99.0	2073	98.7	4696	98.7	0.19	2.76	2.50
	Yes	155	1.0	28	1.3	62	1.3			
Race^: Black or African American	No	14508	96.8	1974	94.0	4536	95.3	<0.001	13.46	7.42
	Yes	483	3.2	127	6.0	222	4.7			
Race^: Native Hawaiian or Pacific Islander	No	14977	99.9	>2090	>99.5	>4747	>99.8	0.013	5.20	0.99
	Yes	14	0.1	**<11**	<0.5	**<11**	<0.2			
Race^: White	No	1070	7.1	228	10.9	464	9.8	<0.001	13.01	9.41
	Yes	13921	92.9	1873	89.1	4294	90.2			
Race^: UTD	No	14589	97.3	2042	97.2	4587	96.4	0.005	0.77	5.23
	Yes	402	2.7	59	2.8	171	3.6			
Hispanic Ethnicity^	Yes	523	3.5	85	4.0	145	3.0	0.099	2.93	2.48
	No / UTD	14468	96.5	2016	96.0	4613	97.0			
Insurance^	Private/HMO/Other	5516	36.8	803	38.2	1812	38.1	<0.001	10.93	11.67
	Medicaid	773	5.2	156	7.4	366	7.7			
	Medicare	6678	44.5	894	42.6	2013	42.3			
	Medicare - Private/HMO/Other	2024	13.5	248	11.8	567	11.9			
Admission Year++ /ranks	2013-2014	557	3.7	87	4.1	152	3.2	0.13	3.23	2.07

Variable	Level	No HF		HFrEF		HFpEF/HFmrEF		P-value+	Std Dif Score* (versus No HF)	
		(N=14991)		(N=2101)		(N=4758)			HFrEF	HFpEF/HFmrEF
	2015	1303	8.7	184	8.8	354	7.4			
	2016	1938	12.9	287	13.7	656	13.8			
	2017	2542	17.0	375	17.8	810	17.0			
	2018	2784	18.6	363	17.3	883	18.6			
	2019	2811	18.8	383	18.2	953	20.0			
	2020	1897	12.7	277	13.2	590	12.4			
	2021	1159	7.7	145	6.9	360	7.6			
Type of Atrial Fibrillation [Among Atrial Fibrillation Patients]	First Detected Atrial Fibrillation	3964	26.4	237	11.3	554	11.6	<0.001	51.07	45.10
	Paroxysmal Atrial Fibrillation	6282	41.9	794	37.8	2087	43.9			
	Persistent Atrial Fibrillation	2448	16.3	553	26.3	1088	22.9			
	Permanent/long standing persistent Atrial Fibrillation	899	6.0	306	14.6	631	13.3			
	Unable to Determine	1398	9.3	211	10.0	398	8.4			
**Medical History**										
Cardiomyopathy	No	14150	94.4	1115	53.1	4116	86.5	<0.001	106.33	27.06
	Yes	841	5.6	986	46.9	642	13.5			
Anemia	No	13295	88.7	1724	82.1	3740	78.6	<0.001	18.85	27.52
	Yes	1696	11.3	377	17.9	1018	21.4			
COPD	No	12759	85.1	1633	77.7	3452	72.6	<0.001	19.08	31.12
	Yes	2232	14.9	468	22.3	1306	27.4			

Variable	Level	No HF (N=14991)		HFrEF (N=2101)		HFpEF/HFmrEF (N=4758)		P-value+	Std Dif Score* (versus No HF) HFrEF	Std Dif Score* (versus No HF) HFpEF/HFmrEF
Coronary Artery Disease	No	10899	72.7	994	47.3	2755	57.9	<0.001	53.67	31.48
	Yes	4092	27.3	1107	52.7	2003	42.1			
Prior MI	No	13762	91.8	1597	76.0	4121	86.6	<0.001	44.00	16.78
	Yes	1229	8.2	504	24.0	637	13.4			
Prior PCI	No	13355	89.1	1612	76.7	3928	82.6	<0.001	33.29	18.81
	Yes	1636	10.9	489	23.3	830	17.4			
Pacemaker	No	13816	92.2	1806	86.0	4084	85.8	<0.001	19.97	20.33
	Yes	1175	7.8	295	14.0	674	14.2			
ICD only	No	14849	99.1	1790	85.2	4636	97.4	<0.001	53.23	12.33
	Yes	142	0.9	311	14.8	122	2.6			
CRT-D	No	14937	99.6	1909	90.9	4673	98.2	<0.001	42.18	13.87
	Yes	54	0.4	192	9.1	85	1.8			
CRT-P	No	14973	99.9	2086	99.3	4730	99.4	<0.001	9.23	7.89
	Yes	18	0.1	15	0.7	28	0.6			
Prior Stroke or TIA	No	12672	84.5	1720	81.9	3812	80.1	<0.001	7.13	11.59
	Yes	2319	15.5	381	18.1	946	19.9			
Diabetes	No	11117	74.2	1365	65.0	3138	66.0	<0.001	20.07	17.99
	Yes	3874	25.8	736	35.0	1620	34.0			
Family History of AF	No	14803	98.7	2089	99.4	4710	99.0	0.014	7.19	2.32
	Yes	188	1.3	12	0.6	48	1.0			

Variable	Level	No HF		HFrEF		HFpEF/HFmrEF		P-value+	Std Dif Score* (versus No HF)	
		(N=14991)		(N=2101)		(N=4758)			HFrEF	HFpEF/HFmrEF
Hypertension	No	3035	20.2	418	19.9	734	15.4	<0.001	0.87	12.61
	Yes	11956	79.8	1683	80.1	4024	84.6			
Left Ventricular Hypertrophy	No	14800	98.7	2051	97.6	4658	97.9	<0.001	8.26	6.43
	Yes	191	1.3	50	2.4	100	2.1			
Liver Disease	No	14868	99.2	2075	98.8	4689	98.5	0.001	4.13	5.95
	Yes	123	0.8	26	1.2	69	1.5			
Mechanical Prosthetic Heart Valve	No	14855	99.1	2044	97.3	4679	98.3	<0.001	13.58	6.69
	Yes	136	0.9	57	2.7	79	1.7			
Mitral Stenosis	No	14905	99.4	2076	98.8	4693	98.6	<0.001	6.60	8.09
	Yes	86	0.6	25	1.2	65	1.4			
Obstructive Sleep Apnea	No	12902	86.1	1735	82.6	3753	78.9	<0.001	9.60	18.99
	Yes	2089	13.9	366	17.4	1005	21.1			
Peripheral Vascular Disease	No	14032	93.6	1897	90.3	4246	89.2	<0.001	12.20	15.63
	Yes	959	6.4	204	9.7	512	10.8			
Prior Hemorrhage	No	14266	95.2	1965	93.5	4349	91.4	<0.001	7.09	15.06
	Yes	725	4.8	136	6.5	409	8.6			
Renal Disease	No	14185	94.6	1855	88.3	4233	89.0	<0.001	22.80	20.72
	Yes	806	5.4	246	11.7	525	11.0			
Dialysis	No	14808	98.8	2062	98.1	4652	97.8	<0.001	5.17	7.74
	Yes	183	1.2	39	1.9	106	2.2			

Variable	Level	No HF		HFrEF		HFpEF/HFmrEF		P-value+	Std Dif Score* (versus No HF)	
		(N=14991)		(N=2101)		(N=4758)			HFrEF	HFpEF/HFmrEF
Rheumatic Heart Disease	No	14944	99.7	2081	99.0	4714	99.1	<0.001	8.06	7.80
	Yes	47	0.3	20	1.0	44	0.9			
Sinus Node Dysfunction	No	14367	95.8	1983	94.4	4405	92.6	<0.001	6.75	13.98
	Yes	624	4.2	118	5.6	353	7.4			
History of cigarette smoking in the past 12 months^	No	14064	93.8	1948	92.7	4487	94.3	0.042	4.38	2.06
	Yes	927	6.2	153	7.3	271	5.7			
Thyroid Disease	No	11971	79.9	1687	80.3	3550	74.6	<0.001	1.10	12.53
	Yes	3020	20.1	414	19.7	1208	25.4			
None	No	14381	95.9	2101	100.0	4758	100.0			
	Yes	610	4.1	0	0.0	0	0.0			
**Labs**										
eGFR (2021 CKD-EPI using NEJM formula)++ /ranks	Median	14822	67.5	2086	55.5	4707	57.0	<0.001	44.36	38.26
	25th		51.4		40.0		40.5			
	75th		83.7		73.1		75.8			
	Mean		66.5		56.8		57.8			
	STD		23.5		24.8		24.4			
	Missing(%)		1.1		0.7		1.1			
**Other Risk Factors**										
CHA^2^DS^2^VASc score++ /ranks	Median	14844	4.0	2088	5.0	4722	5.0	<0.001	73.87	94.42
	25th		3.0		4.0		5.0			
	75th		5.0		6.0		6.0			

Variable	Level	No HF (N=14991)		HFrEF (N=2101)		HFpEF/HFmrEF (N=4758)		P-value+	Std Dif Score* (versus No HF)	
									HFrEF	HFpEF/HFmrEF
	Mean		4.2		5.2		5.5			
	STD		1.4		1.4		1.4			
	Missing(%)		1.0		0.6		0.8			
LVEF++ /ranks	Median	14991	58.0	2101	30.0	4758	57.0	<0.001	239.64	7.50
	25th		52.0		23.0		50.0			
	75th		63.0		35.0		62.0			
	Mean		56.0		29.4		56.6			
	STD		13.2		7.8		7.8			
	Missing(%)		0.0		0.0		0.0			
Labile INR	Missing	2245	15.0	315	15.0	649	13.6	<0.001	13.36	9.95
	No	12453	83.1	1701	81.0	3943	82.9			
	Yes	293	2.0	85	4.0	166	3.5			
**<u>Medications at Discharge</u>**										
ACEi Prescribed?#	Yes	3510	23.4	726	34.6	986	20.7	<0.001	73.90	9.07
	No	10188	68.0	747	35.6	3258	68.5			
	NC	1293	8.6	628	29.9	514	10.8			
ARB Prescribed?#	Yes	2466	16.4	376	17.9	791	16.6	<0.001	59.13	6.23
	No	11547	77.0	1160	55.2	3581	75.3			
	NC	978	6.5	565	26.9	386	8.1			
ARNi Prescribed? (collected beginning in 2016)#	Yes	87	0.7	146	8.0	38	0.9	<0.001	54.86	4.33
	No	12114	92.3	1334	72.9	3875	91.1			
	NC	930	7.1	350	19.1	339	8.0			

Variable	Level	No HF		HFrEF		HFpEF/HFmrEF		P-value+	Std Dif Score* (versus No HF)	
		(N=14991)		(N=2101)		(N=4758)			HFrEF	HFpEF/HFmrEF
Beta-Blocker Prescribed?#	Yes	9779	65.2	1781	84.8	3355	70.5	<0.001	63.18	13.60
	No	4324	28.8	127	6.0	1090	22.9			
	NC	888	5.9	193	9.2	313	6.6			
Statin Prescribed?#	Yes	8663	57.8	1375	65.4	2940	61.8	<0.001	21.47	13.29
	No	5379	35.9	550	26.2	1430	30.1			
	NC	949	6.3	176	8.4	388	8.2			
Antiarrhythmic Prescribed?#	Yes	6245	41.7	918	43.7	1859	39.1	0.001	6.69	6.00
	No	8683	57.9	1166	55.5	2868	60.3			
	NC	63	0.4	17	0.8	31	0.7			
Antiplatelet Prescribed?#	Yes	6510	43.4	1104	52.5	2198	46.2	<0.001	18.56	6.29
	No	7478	49.9	866	41.2	2224	46.7			
	NC	1003	6.7	131	6.2	336	7.1			
DOAC Prescribed?#	Yes	9264	61.8	1173	55.8	2561	53.8	<0.001	14.85	17.00
	No	3692	24.6	656	31.2	1499	31.5			
	NC	2035	13.6	272	12.9	698	14.7			
Anticoagulation: Warfarin Prescribed?#	Yes	2811	18.8	555	26.4	1309	27.5	<0.001	18.53	22.39
	No	10145	67.7	1274	60.6	2751	57.8			
	NC	2035	13.6	272	12.9	698	14.7			
Ca Channel Blocker Prescribed?#	Yes	5013	33.4	244	11.6	1657	34.8	<0.001	54.18	3.08
	No	9310	62.1	1744	83.0	2884	60.6			
	NC	668	4.5	113	5.4	217	4.6			
**Discharge Disposition**										

Variable	Level	No HF		HFrEF		HFpEF/HFmrEF		P-value+	Std Dif Score* (versus No HF)	
		(N=14991)		(N=2101)		(N=4758)			HFrEF	HFpEF/HFmrEF
Discharge Disposition	1 - Home	12414	82.8	1636	77.9	3675	77.2	<0.001	15.75	14.87
	2 - Hospice - Home	135	0.9	48	2.3	77	1.6			
	3 - Hospice - Health Care Facility	129	0.9	34	1.6	51	1.1			
	4 - Acute Care Facility	163	1.1	23	1.1	47	1.0			
	5 - Other Health Care Facility	2150	14.3	360	17.1	908	19.1			
**<u>Hospital Characteristics</u>**										
Academic/Teaching Hospital	Missing	716	4.8	112	5.3	189	4.0	<0.001	14.62	7.44
	No	2796	18.7	281	13.4	764	16.1			
	Yes	11479	76.6	1708	81.3	3805	80.0			
Rural Location	Missing	716	4.8	112	5.3	189	4.0	<0.001	8.83	6.53
	No	13031	86.9	1862	88.6	4251	89.3			
	Yes	1244	8.3	127	6.0	318	6.7			
Hospital Size (Number of Beds)	Missing	716	4.8	112	5.3	189	4.0	<0.001	20.47	12.58
	25-99	490	3.3	38	1.8	111	2.3			
	100-199	2246	15.0	282	13.4	643	13.5			
	200-299	1602	10.7	170	8.1	405	8.5			
	300-399	2802	18.7	341	16.2	980	20.6			
	400-499	2352	15.7	450	21.4	860	18.1			
	500+	4783	31.9	708	33.7	1570	33.0			
Region	Northeast	4581	30.6	572	27.2	1431	30.1	<0.001	22.58	11.46
	Midwest	3236	21.6	418	19.9	1057	22.2			

Variable	Level	No HF (N=14991)		HFrEF (N=2101)		HFpEF/HFmrEF (N=4758)		P-value+	Std Dif Score* (versus No HF) HFrEF	HFpEF/HFmrEF
	South	5778	38.5	757	36.0	1672	35.1			
	West	1396	9.3	354	16.8	598	12.6			
Patient Volume Discharges	Missing	392	2.6	78	3.7	174	3.7	<0.001	7.72	6.63
	0 - 100 Discharges	12773	85.2	1717	81.7	3939	82.8			
	101 - 300 Discharges	1126	7.5	183	8.7	356	7.5			
	300+ Discharges	700	4.7	123	5.9	289	6.1			

ACEi: angiotensin-converting enzyme inhibitor; AF: atrial fibrillation; AIAN: American Indian or Alaska Native; ARB: Angiotensin II receptor blocker; ARNi: Angiotensin receptor neprilysin inhibitor; BMI: body mass index; CAD: coronary artery disease; CCA: calcium channel blocker; COPD: chronic obstructive pulmonary disease; DOAC: direct oral anticoagulant; HF: heart failure; HFmrEF: heart failure with mid-range ejection fraction; HFpEF: heart failure with preserved ejection fraction; HFrEF: heart failure with reduced ejection fraction; LVEF: left ventricular ejection fraction; OSA: obstructive sleep apnea; PAD: peripheral artery disease; TIA: transient ischemic attack; UTD: unable to determine
Categorical variables are reported as N (%)

+ Note: P-values do not correspond to the table exactly as it is presented here. More appropriately, p-values were calculated by comparing only non-missing row values.
+ P-values are based on Pearson chi-square tests for all categorical row variables, unless indicated as /Fisher for Fisher-exact p-values.
++ P-values are based on chi-square rank based group means score statistics for all continuous/ordinal row variables (designated by ++).
++ This is equivalent to Kruskal-Wallis tests.
All tests treat the column variable as nominal.

* Standardized difference scores measure the effect size between indicated group and reference group: No HF.
* /ranks indicates standardized difference score was calculated using rank statistics for the row variable.
* All Standardized difference scores have been multiplied by 100.

^Missing values were imputed to most frequent category to avoid cell size <11.
#Missing values were imputed to No taking medication to avoid cell size <11.

Table S3. Patient characteristics by body mass index categories

Variable	Level	Underweight/ Normal (BMI<24.9) (N=6452)		Overweight (BMI 25-29.9) (N=6990)		Obesity (BMI≥30) (N=8408)		P-value+	Std Dif Score* (versus Underweight/Normal)	
									Overweight	Obesity
**<u>Demographics</u>**										
Age++ /ranks	Median	6452	82.0	6990	78.0	8408	74.0	<0.001	34.37	84.53
	25th		75.0		72.0		69.0			
	75th		88.0		85.0		79.0			
	Mean		81.3		78.5		74.8			
	STD		8.2		8.0		7.0			
	Missing(%)		0.0		0.0		0.0			
Age++ /ranks	<75	1509	23.4	2441	34.9	4506	53.6	<0.001	25.58	65.30
	>=75	4943	76.6	4549	65.1	3902	46.4			
Sex	Male	2411	37.4	3603	51.5	3723	44.3	<0.001	28.82	14.10
	Female	4041	62.6	3387	48.5	4685	55.7			
BMI (kg/m^^{2}^)++ /ranks	Median	6452	22.4	6990	27.4	8408	34.4	<0.001	346.14	343.45
	25th		20.5		26.2		31.9			
	75th		23.8		28.6		38.5			
	Mean		21.9		27.4		36.3			
	STD		2.4		1.4		8.3			
	Missing(%)		0.0		0.0		0.0			
Race^: American Indian or Alaska Native	No	6435	99.7	6974	99.8	8387	99.8	0.92	0.70	0.27
	Yes	17	0.3	16	0.2	21	0.2			
Race^: Asian	No	6323	98.0	6909	98.8	8373	99.6	<0.001	6.75	14.53
	Yes	129	2.0	81	1.2	35	0.4			

Variable	Level	Underweight/ Normal (BMI<24.9) (N=6452)		Overweight (BMI 25-29.9) (N=6990)		Obesity (BMI≥30) (N=8408)		P-value+	Std Dif Score* (versus Underweight/Normal)	
									**Overweight**	**Obesity**
Race^: Black or African American	No	6223	96.5	6759	96.7	8036	95.6	0.001	1.34	4.47
	Yes	229	3.5	231	3.3	372	4.4			
Race^: Native Hawaiian or Pacific Islander	No	>6441	>99.8	>6979	>99.8	8393	99.8	0.16	1.23	1.85
	Yes	**<11**	<0.2	**<11**	<0.2	15	0.2			
Race^: White	No	560	8.7	534	7.6	668	7.9	0.076	3.80	2.66
	Yes	5892	91.3	6456	92.4	7740	92.1			
Race^: UTD	No	6268	97.1	6779	97.0	8171	97.2	0.74	0.99	0.20
	Yes	184	2.9	211	3.0	237	2.8			
Hispanic Ethnicity^	Yes	210	3.3	236	3.4	307	3.7	0.39	0.68	2.17
	No / UTD	6242	96.7	6754	96.6	8101	96.3			
Insurance^	Private/HMO/Other	2331	36.1	2625	37.6	3175	37.8	0.003	4.03	5.20
	Medicaid	389	6.0	370	5.3	536	6.4			
	Medicare	2857	44.3	3046	43.6	3682	43.8			
	Medicare - Private/HMO/Other	875	13.6	949	13.6	1015	12.1			
Admission Year++ /ranks	2013-2014	275	4.3	237	3.4	284	3.4	<0.001	5.32	6.98
	2015	604	9.4	536	7.7	701	8.3			
	2016	862	13.4	986	14.1	1033	12.3			
	2017	1112	17.2	1172	16.8	1443	17.2			
	2018	1167	18.1	1289	18.4	1574	18.7			

Variable	Level	Underweight/ Normal (BMI<24.9) (N=6452)		Overweight (BMI 25-29.9) (N=6990)		Obesity (BMI≥30) (N=8408)		P-value+	Std Dif Score* (versus Underweight/Normal)	
									Overweight	Obesity
	2019	1154	17.9	1370	19.6	1623	19.3			
	2020	840	13.0	853	12.2	1071	12.7			
	2021	438	6.8	547	7.8	679	8.1			
Type of Atrial Fibrillation [Among Atrial Fibrillation Patients]	First Detected Atrial Fibrillation	1499	23.2	1466	21.0	1790	21.3	<0.001	12.47	17.36
	Paroxysmal Atrial Fibrillation	2749	42.6	2929	41.9	3485	41.4			
	Persistent Atrial Fibrillation	969	15.0	1334	19.1	1786	21.2			
	Permanent/long standing Persistent Atrial Fibrillation	631	9.8	568	8.1	637	7.6			
	Unable to Determine	604	9.4	693	9.9	710	8.4			
**<u>Medical History</u>**										
Cardiomyopathy	No	5735	88.9	6166	88.2	7480	89.0	0.29	2.12	0.24
	Yes	717	11.1	824	11.8	928	11.0			
Anemia	No	5436	84.3	6061	86.7	7262	86.4	<0.001	6.98	5.98
	Yes	1016	15.7	929	13.3	1146	13.6			
COPD	No	5197	80.5	5847	83.6	6800	80.9	<0.001	8.09	0.83
	Yes	1255	19.5	1143	16.4	1608	19.1			
Coronary Artery Disease	No	4532	70.2	4617	66.1	5499	65.4	<0.001	9.00	10.37
	Yes	1920	29.8	2373	33.9	2909	34.6			

Variable	Level	Underweight/ Normal (BMI<24.9) (N=6452)		Overweight (BMI 25-29.9) (N=6990)		Obesity (BMI≥30) (N=8408)		P-value+	Std Dif Score* (versus Underweight/Normal)	
									Overweight	Obesity
Prior MI	No	5775	89.5	6188	88.5	7517	89.4	0.12	3.14	0.34
	Yes	677	10.5	802	11.5	891	10.6			
Prior PCI	No	5712	88.5	6004	85.9	7179	85.4	<0.001	7.90	9.36
	Yes	740	11.5	986	14.1	1229	14.6			
Pacemaker	No	5746	89.1	6277	89.8	7683	91.4	<0.001	2.41	7.81
	Yes	706	10.9	713	10.2	725	8.6			
ICD only	No	6286	97.4	6775	96.9	8214	97.7	0.012	3.04	1.72
	Yes	166	2.6	215	3.1	194	2.3			
CRT-D	No	6352	98.5	6876	98.4	8291	98.6	0.46	0.65	1.32
	Yes	100	1.5	114	1.6	117	1.4			
CRT-P	No	6430	99.7	6971	99.7	8388	99.8	0.49	1.25	1.92
	Yes	22	0.3	19	0.3	20	0.2			
Prior Stroke or TIA	No	5195	80.5	5826	83.3	7183	85.4	<0.001	7.36	13.10
	Yes	1257	19.5	1164	16.7	1225	14.6			
Diabetes	No	5328	82.6	5228	74.8	5064	60.2	<0.001	19.10	51.05
	Yes	1124	17.4	1762	25.2	3344	39.8			
Family History of AF	No	6391	99.1	6917	99.0	8294	98.6	0.044	1.00	3.85
	Yes	61	0.9	73	1.0	114	1.4			
Heart Failure	No	4529	70.2	4935	70.6	5527	65.7	<0.001	0.89	9.57
	Yes	1923	29.8	2055	29.4	2881	34.3			

Variable	Level	Underweight/ Normal (BMI<24.9) (N=6452)		Overweight (BMI 25-29.9) (N=6990)		Obesity (BMI≥30) (N=8408)		P-value+	Std Dif Score* (versus Underweight/Normal)	
									Overweight	Obesity
Hypertension	No	1556	24.1	1388	19.9	1243	14.8	<0.001	10.30	23.74
	Yes	4896	75.9	5602	80.1	7165	85.2			
Left Ventricular Hypertrophy	No	6362	98.6	6876	98.4	8271	98.4	0.441	1.93	1.92
	Yes	90	1.4	114	1.6	137	1.6			
Liver Disease	No	6389	99.0	6926	99.1	8317	98.9	0.572	0.63	1.05
	Yes	63	1.0	64	0.9	91	1.1			
Mechanical Prosthetic Heart Valve	No	6356	98.5	6901	98.7	8321	99.0	0.046	1.84	4.06
	Yes	96	1.5	89	1.3	87	1.0			
Mitral Stenosis	No	6381	98.9	6936	99.2	8357	99.4	0.004	3.40	5.37
	Yes	71	1.1	54	0.8	51	0.6			
Obstructive Sleep Apnea	No	6100	94.5	6201	88.7	6089	72.4	<0.001	21.17	62.42
	Yes	352	5.5	789	11.3	2319	27.6			
Peripheral Vascular Disease	No	5964	92.4	6450	92.3	7761	92.3	0.93	0.61	0.50
	Yes	488	7.6	540	7.7	647	7.7			
Prior Hemorrhage	No	6084	94.3	6588	94.2	7908	94.1	0.79	0.20	1.04
	Yes	368	5.7	402	5.8	500	5.9			
Renal Disease	No	5996	92.9	6508	93.1	7769	92.4	0.21	0.67	2.04
	Yes	456	7.1	482	6.9	639	7.6			
Dialysis	No	6350	98.4	6893	98.6	8279	98.5	0.62	1.60	0.38

Variable	Level	Underweight/ Normal (BMI<24.9) (N=6452)		Overweight (BMI 25-29.9) (N=6990)		Obesity (BMI≥30) (N=8408)		P-value+	Std Dif Score* (versus Underweight/Normal) Overweight	Obesity
	Yes	102	1.6	97	1.4	129	1.5			
Rheumatic Heart Disease	No	6403	99.2	6957	99.5	8379	99.7	0.003	3.67	5.60
	Yes	49	0.8	33	0.5	29	0.3			
Sinus Node Dysfunction	No	6105	94.6	6621	94.7	8029	95.5	0.025	0.44	4.02
	Yes	347	5.4	369	5.3	379	4.5			
History of cigarette smoking in the past 12 months^	No	5949	92.2	6581	94.1	7969	94.8	<0.001	7.72	10.45
	Yes	503	7.8	409	5.9	439	5.2			
Thyroid Disease	No	5011	77.7	5595	80.0	6602	78.5	0.003	5.82	2.07
	Yes	1441	22.3	1395	20.0	1806	21.5			
None	No	6209	96.2	6772	96.9	8259	98.2	<0.001	3.55	12.18
	Yes	243	3.8	218	3.1	149	1.8			
**Labs**										
eGFR (2021 CKD-EPI using NEJM formula)++ /ranks	Median	6385	63.5	6919	64.6	8311	64.6	0.20	1.95	2.98
	25th		46.9		47.8		48.2			
	75th		82.6		81.5		81.0			
	Mean		63.2		63.8		63.9			
	STD		24.0		24.3		24.1			
	Missing(%)		1.0		1.0		1.2			
**Other Risk Factors**										

Variable	Level	Underweight/ Normal (BMI<24.9) (N=6452)		Overweight (BMI 25-29.9) (N=6990)		Obesity (BMI≥30) (N=8408)		P-value+	Std Dif Score* (versus Underweight/Normal)	
									Overweight	Obesity
CHA^$_{2}$^DS^$_{2}$^VASc score++ /ranks	Median	6387	5.0	6923	4.0	8344	4.0	<0.001	11.36	7.09
	25th		4.0		3.0		3.0			
	75th		6.0		5.0		6.0			
	Mean		4.7		4.5		4.6			
	STD		1.5		1.5		1.5			
	Missing(%)		1.0		1.0		0.8			
LVEF++ /ranks	Median	6452	57.0	6990	56.0	8408	56.0	0.008	5.29	2.48
	25th		48.0		48.0		50.0			
	75th		63.0		62.0		62.0			
	Mean		53.7		53.3		53.7			
	STD		15.5		14.8		12.4			
	Missing(%)		0.0		0.0		0.0			
Labile INR	Missing	946	14.7	1036	14.8	1227	14.6	0.96	0.41	0.14
	No	5347	82.9	5778	82.7	6972	82.9			
	Yes	159	2.5	176	2.5	209	2.5			
**<u>Medications at Discharge</u>**										
ACEi Prescribed?#	Yes	1309	20.3	1660	23.7	2253	26.8	<0.001	9.08	15.81
	No	4338	67.2	4572	65.4	5283	62.8			
	NC	805	12.5	758	10.8	872	10.4			
ARB Prescribed?#	Yes	794	12.3	1092	15.6	1747	20.8	<0.001	10.33	24.09
	No	4984	77.2	5273	75.4	6031	71.7			
	NC	674	10.4	625	8.9	630	7.5			

Variable	Level	Underweight/ Normal (BMI<24.9) (N=6452)		Overweight (BMI 25-29.9) (N=6990)		Obesity (BMI≥30) (N=8408)		P-value+	Std Dif Score* (versus Underweight/Normal)	
									Overweight	Obesity
ARNi Prescribed? (collected beginning in 2016)#	Yes	58	1.0	84	1.4	129	1.7	0.018	2.96	5.99
	No	5044	90.5	5596	90.0	6683	90.0			
	NC	471	8.5	537	8.6	611	8.2			
Beta-Blocker Prescribed?#	Yes	4276	66.3	4788	68.5	5851	69.6	0.001	4.91	7.11
	No	1733	26.9	1773	25.4	2035	24.2			
	NC	443	6.9	429	6.1	522	6.2			
Statin Prescribed?#	Yes	3268	50.7	4198	60.1	5512	65.6	<0.001	19.06	30.77
	No	2671	41.4	2319	33.2	2369	28.2			
	NC	513	8.0	473	6.8	527	6.3			
Antiarrhythmic Prescribed?#	Yes	2292	35.5	2911	41.6	3819	45.4	<0.001	12.62	20.33
	No	4120	63.9	4043	57.8	4554	54.2			
	NC	40	0.6	36	0.5	35	0.4			
Antiplatelet Prescribed?#	Yes	2818	43.7	3157	45.2	3837	45.6	<0.001	5.51	8.20
	No	3122	48.4	3373	48.3	4073	48.4			
	NC	512	7.9	460	6.6	498	5.9			
DOAC Prescribed?#	Yes	3459	53.6	4187	59.9	5352	63.7	<0.001	19.78	31.02
	No	1692	26.2	1898	27.2	2257	26.8			
	NC	1301	20.2	905	12.9	799	9.5			
Anticoagulation: Warfarin Prescribed?#	Yes	1251	19.4	1511	21.6	1913	22.8	<0.001	19.54	30.40
	No	3900	60.4	4574	65.4	5696	67.7			
	NC	1301	20.2	905	12.9	799	9.5			
	Yes	1977	30.6	2113	30.2	2824	33.6	<0.001	3.42	8.37

Variable	Level	Underweight/ Normal (BMI<24.9) (N=6452)		Overweight (BMI 25-29.9) (N=6990)		Obesity (BMI≥30) (N=8408)		P-value+	Std Dif Score* (versus Underweight/Normal)	
									Overweight	Obesity
Ca Channel Blocker Prescribed?#	No	4133	64.1	4554	65.2	5251	62.5			
	NC	342	5.3	323	4.6	333	4.0			
**Discharge Disposition**										
Discharge Disposition	1 - Home	4813	74.6	5736	82.1	7176	85.3	<0.001	19.09	28.69
	2 - Hospice - Home	137	2.1	71	1.0	52	0.6			
	3 - Hospice - Health Care Facility	104	1.6	65	0.9	45	0.5			
	4 - Acute Care Facility	76	1.2	74	1.1	83	1.0			
	5 - Other Health Care Facility	1322	20.5	1044	14.9	1052	12.5			
**Hospital Characteristics**										
Academic/Teaching Hospital	Missing	264	4.1	336	4.8	417	5.0	0.70	1.10	0.25
	No	1147	17.8	1205	17.2	1489	17.7			
	Yes	5041	78.1	5449	78.0	6502	77.3			
Rural Location	Missing	264	4.1	336	4.8	417	5.0	<0.001	5.45	7.78
	No	5605	86.9	6129	87.7	7410	88.1			
	Yes	583	9.0	525	7.5	581	6.9			
Hospital Size (Number of Beds)	Missing	264	4.1	336	4.8	417	5.0	<0.001	8.94	13.35
	25-99	218	3.4	198	2.8	223	2.7			
	100-199	991	15.4	998	14.3	1182	14.1			
	200-299	746	11.6	679	9.7	752	8.9			

Variable	Level	Underweight/ Normal (BMI<24.9) (N=6452)		Overweight (BMI 25-29.9) (N=6990)		Obesity (BMI≥30) (N=8408)		P-value+	Std Dif Score* (versus Underweight/Normal)	
									Overweight	Obesity
	300-399	1195	18.5	1326	19.0	1602	19.1			
	400-499	1122	17.4	1183	16.9	1357	16.1			
	500+	1916	29.7	2270	32.5	2875	34.2			
Region	Northeast	1896	29.4	2142	30.6	2546	30.3	<0.001	5.21	10.72
	Midwest	1321	20.5	1480	21.2	1910	22.7			
	South	2434	37.7	2604	37.3	3169	37.7			
	West	801	12.4	764	10.9	783	9.3			
Patient Volume Discharges	Missing	176	2.7	193	2.8	275	3.3	0.001	6.40	5.67
	0 - 100 Discharges	5538	85.8	5864	83.9	7027	83.6			
	101 - 300 Discharges	446	6.9	532	7.6	687	8.2			
	300+ Discharges	292	4.5	401	5.7	419	5.0			

ACEi: angiotensin-converting enzyme inhibitor; AF: atrial fibrillation; AIAN: American Indian or Alaska Native; ARB: Angiotensin II receptor blocker; ARNi: Angiotensin receptor neprilysin inhibitor; BMI: body mass index; CAD: coronary artery disease; CCA: calcium channel blocker; COPD: chronic obstructive pulmonary disease; DOAC: direct oral anticoagulant; HF: heart failure; HFmrEF: heart failure with mid-range ejection fraction; HFpEF: heart failure with preserved ejection fraction; HFrEF: heart failure with reduced ejection fraction; LVEF: left ventricular ejection fraction; OSA: obstructive sleep apnea; PAD: peripheral artery disease; TIA: transient ischemic attack; UTD: unable to determine
Categorical variables are reported as N (%)

+ Note: P-values do not correspond to the table exactly as it is presented here. More appropriately, p-values were calculated by comparing only non-missing row values.
+ P-values are based on Pearson chi-square tests for all categorical row variables, unless indicated as /Fisher for Fisher-exact p-values.
++ P-values are based on chi-square rank based group means score statistics for all continuous/ordinal row variables (designated by ++).
++ This is equivalent to Kruskal-Wallis tests.
All tests treat the column variable as nominal.

* Standardized difference scores measure the effect size between indicated group and reference group: No HF.
* /ranks indicates standardized difference score was calculated using rank statistics for the row variable.
* All Standardized difference scores have been multiplied by 100.

^Missing values were imputed to most frequent category to avoid cell size <11.
#Missing values were imputed to No taking medication to avoid cell size <11.

Table S4. Adjusted association of body mass index with outcomes according to heart failure status

		No HF		HFrEF		HFpEF/HFmrEF		
Outcome	Effect	HR (95% CI)	P-value	HR (95% CI)	P-value	HR (95% CI)	P-value	Interaction P-value+
Mortality within 30 days	BMI<30 per 1 unit increase	0.93 (0.91, 0.95)	<0.001	0.96 (0.91, 1.01)	0.124	0.91 (0.87, 0.95)	<0.001	0.27
	BMI>=30 per 1 unit increase	1.02 (0.99, 1.05)	0.158	1.02 (0.95, 1.10)	0.567	1.03 (0.99, 1.07)	0.15	
Mortality within 1 year	BMI<30 per 1 unit increase	0.94 (0.92, 0.95)	<0.001	0.96 (0.93, 0.99)	0.003	0.93 (0.92, 0.95)	<0.001	0.013
	BMI>=30 per 1 unit increase	1.01 (1.00, 1.03)	0.162	1.00 (0.97, 1.03)	0.978	1.01 (1.00, 1.03)	0.14	
Cardiovascular hospitalization* within 30 days	BMI<30 per 1 unit increase	1.02 (1.00, 1.04)	0.043	0.97 (0.94, 1.01)	0.107	1.00 (0.97, 1.03)	0.81	0.11
	BMI>=30 per 1 unit increase	0.99 (0.97, 1.01)	0.179	1.00 (0.97, 1.04)	0.786	0.98 (0.95, 1.01)	0.13	
Cardiovascular hospitalization* within 1 year	BMI<30 per 1 unit increase	1.00 (0.99, 1.01)	0.423	0.98 (0.96, 1.00)	0.046	0.99 (0.97, 1.00)	0.11	0.20
	BMI>=30 per 1 unit increase	1.00 (0.99, 1.01)	0.434	1.01 (0.99, 1.03)	0.439	1.00 (0.99, 1.01)	0.99	
Ischemic Stroke/TIA/SE* within 30 days	BMI<30 per 1 unit increase	1.03 (0.98, 1.09)	0.261	1.15 (0.94, 1.40)	0.176	0.98 (0.66, 1.47)	0.93	0.72
	BMI>=30 per 1 unit increase	0.90 (0.83, 0.98)	0.017	0.88 (0.73, 1.07)	0.201	1.08 (0.63, 1.88)	0.77	
Ischemic Stroke/TIA/SE* within 1 year	BMI<30 per 1 unit increase	0.98 (0.95, 1.02)	0.374	0.97 (0.88, 1.08)	0.596	0.98 (0.92, 1.05)	0.61	0.66

		No HF		HFrEF		HFpEF/HFmrEF		
**Outcome**	**Effect**	**HR (95% CI)**	**P-value**	**HR (95% CI)**	**P-value**	**HR (95% CI)**	**P-value**	**Interaction P-value+**
	BMI>=30 per 1 unit increase	0.96 (0.92, 1.01)	0.113	0.98 (0.92, 1.05)	0.647	0.94 (0.87, 1.00)	0.067	
Myocardial infarction* within 30 days	BMI<30 per 1 unit increase	1.01 (0.92, 1.11)	0.862	0.67 (0.50, 0.89)	0.006	0.85 (0.73, 0.98)	0.029	0.039
	BMI>=30 per 1 unit increase	1.01 (0.91, 1.11)	0.910	1.05 (0.72, 1.52)	0.803	1.08 (0.96, 1.22)	0.19	
Myocardial infarction* within 1 year	BMI<30 per 1 unit increase	0.94 (0.90, 0.98)	0.004	0.91 (0.84, 0.99)	0.028	0.94 (0.87, 1.01)	0.11	0.37
	BMI>=30 per 1 unit increase	1.01 (0.95, 1.06)	0.839	0.96 (0.88, 1.06)	0.418	1.06 (1.02, 1.10)	0.006	

95% CI: 95% confidence interval; BMI: body mass index; HF: heart failure; HFmrEF: heart failure with mid-range ejection fraction; HFpEF: heart failure with preserved ejection fraction; HFrEF: heart failure with reduced ejection fraction; HR: hazard ration; SE: systemic embolism; TIA: transient ischemic attack

*Cause-Specific Cox model with censoring for mortality.

+Interaction P-value between BMI categories and HF status

Table S5. Sensitivity analysis- Adjusted association of body mass index with outcomes according to heart failure status after excluding patients underweight or with extreme body mass index

		No HF		HFrEF		HFpEF/HFmrEF	
**Outcome**	**Effect**	**HR (95% CI)**	**P-value**	**HR (95% CI)**	**P-value**	**HR (95% CI)**	**P-value**
Mortality within 30 days	BMI<30 per 1 unit increase	0.95 (0.92, 0.98)	0.001	0.97 (0.92, 1.03)	0.37	0.92 (0.87, 0.97)	0.002
	BMI>=30 per 1 unit increase	1.01 (0.98, 1.05)	0.47	1.01 (0.94, 1.08)	0.89	1.03 (0.98, 1.07)	0.26
Mortality within 1 year	BMI<30 per 1 unit increase	0.94 (0.93, 0.95)	<0.001	0.96 (0.94, 0.99)	0.008	0.94 (0.92, 0.97)	<0.001
	BMI>=30 per 1 unit increase	1.00 (0.99, 1.02)	0.78	0.99 (0.96, 1.02)	0.37	1.01 (0.99, 1.03)	0.44
Cardiovascular hospitalization* within 30 days	BMI<30 per 1 unit increase	1.03 (1.01, 1.05)	0.011	0.97 (0.94, 1.01)	0.13	0.99 (0.96, 1.03)	0.76
	BMI>=30 per 1 unit increase	0.99 (0.96, 1.01)	0.27	1.01 (0.98, 1.05)	0.50	0.98 (0.95, 1.00)	0.094
Cardiovascular hospitalization* within 1 year	BMI<30 per 1 unit increase	1.01 (0.99, 1.02)	0.22	0.97 (0.95, 1.00)	0.023	0.99 (0.97, 1.00)	0.11
	BMI>=30 per 1 unit increase	1.00 (0.98, 1.01)	0.55	1.02 (1.00, 1.04)	0.11	1.00 (0.99, 1.01)	0.94
Ischemic Stroke/TIA/SE* within 30 days	BMI<30 per 1 unit increase	1.02 (0.96, 1.10)	0.50	1.18 (0.95, 1.46)	0.13	0.96 (0.61, 1.54)	0.88
	BMI>=30 per 1 unit increase	0.92 (0.84, 1.00)	0.060	0.91 (0.78, 1.07)	0.27	1.10 (0.55, 2.20)	0.78
	BMI<30 per 1 unit increase	1.00 (0.97, 1.04)	0.94	1.04 (0.95, 1.15)	0.38	0.99 (0.92, 1.06)	0.73

		No HF		HFrEF		HFpEF/HFmrEF	
**Outcome**	**Effect**	**HR (95% CI)**	**P-value**	**HR (95% CI)**	**P-value**	**HR (95% CI)**	**P-value**
Ischemic Stroke/TIA/SE* within 1 year	BMI>=30 per 1 unit increase	0.94 (0.90, 0.98)	0.005	0.99 (0.92, 1.07)	0.83	0.93 (0.86, 1.01)	0.09
Myocardial infarction* within 30 days	BMI<30 per 1 unit increase	0.98 (0.87, 1.10)	0.73	0.51 (0.31, 0.82)	0.006	0.81 (0.67, 0.97)	0.02
	BMI>=30 per 1 unit increase	0.98 (0.87, 1.11)	0.78	1.23 (0.72, 2.09)	0.46	1.13 (0.97, 1.32)	0.12
Myocardial infarction* within 1 year	BMI<30 per 1 unit increase	0.92 (0.87, 0.97)	0.001	0.85 (0.76, 0.96)	0.008	0.94 (0.86, 1.03)	0.21
	BMI>=30 per 1 unit increase	1.02 (0.96, 1.08)	0.60	0.99 (0.90, 1.09)	0.89	1.09 (1.03, 1.15)	0.001

Patients with body mass index <18.5 or >50 were excluded. BMI >50 was selected as the upper cut-off because the overall 99th percentile was 50.8.

95% CI: 95% confidence interval; BMI: body mass index; HF: heart failure; HFmrEF: heart failure with mid-range ejection fraction; HFpEF: heart failure with preserved ejection fraction; HFrEF: heart failure with reduced ejection fraction; HR: hazard ration; SE: systemic embolism; TIA: transient ischemic attack

*Cause-Specific Cox model with censoring for mortality.

Figure S1. Spline plots assessing the unadjusted association of BMI on death within 30 days in AF patients without HF (Panel A), with HFrEF (Panel B) and HFpEF/HFmrEF (Panel C)

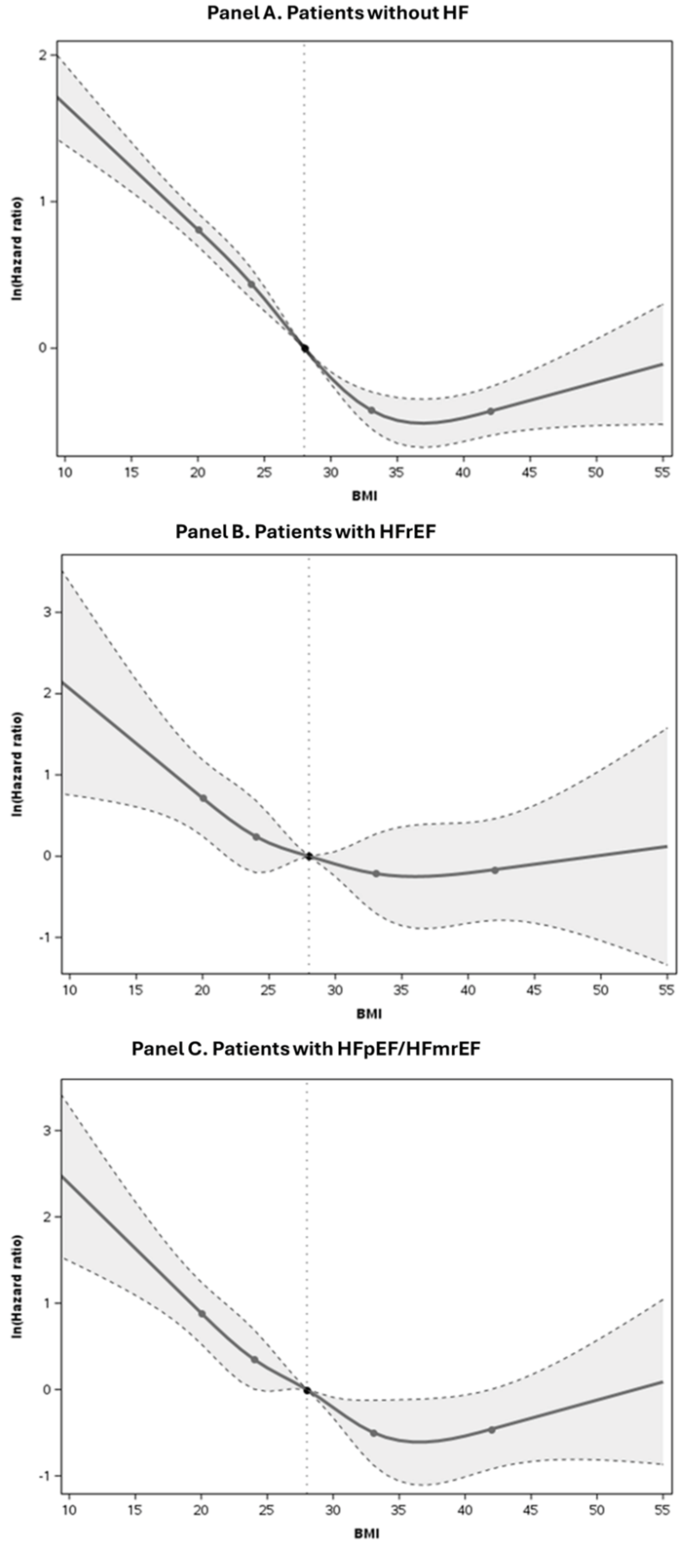

AF: atrial fibrillation; BMI: body mass index; HF: heart failure; HFpEF: heart failure with preserved ejection fraction; HFmrEF: heart failure with mid-range ejection fraction; HFrEF: heart failure with reduced ejection fraction

Figure S2. Cumulative incidence curves for ischemic stroke, transient ischemic attack, and systematic embolism across body mass index categories in AF patients without HF (Panel A), with HFrEF (Panel B) and HFpEF/HFmrEF (Panel C)

Panel A. Patients without HF

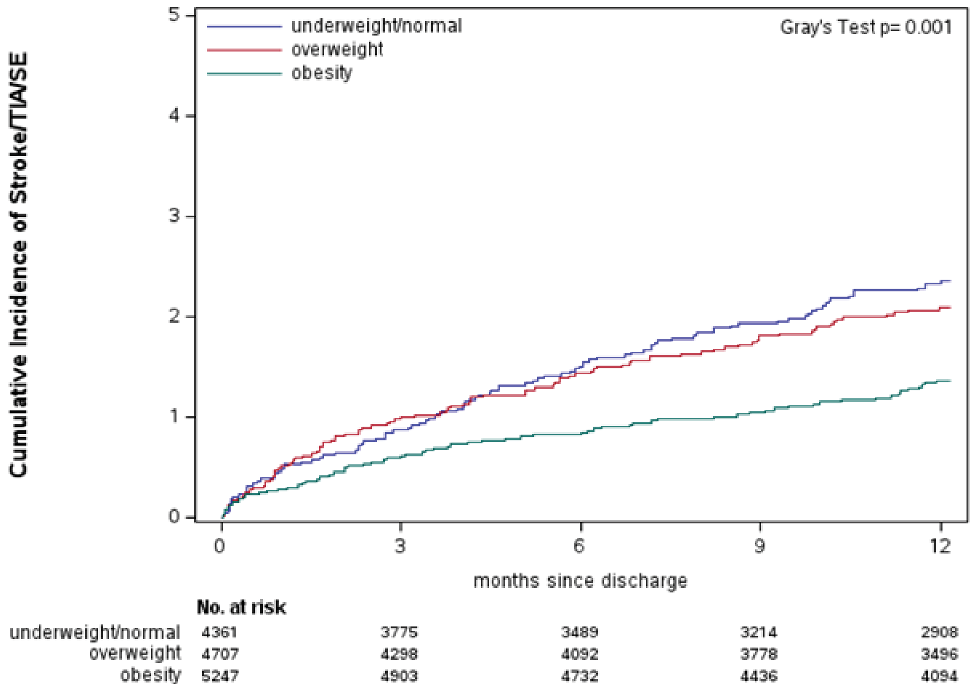

Panel B. Patients with HFrEF

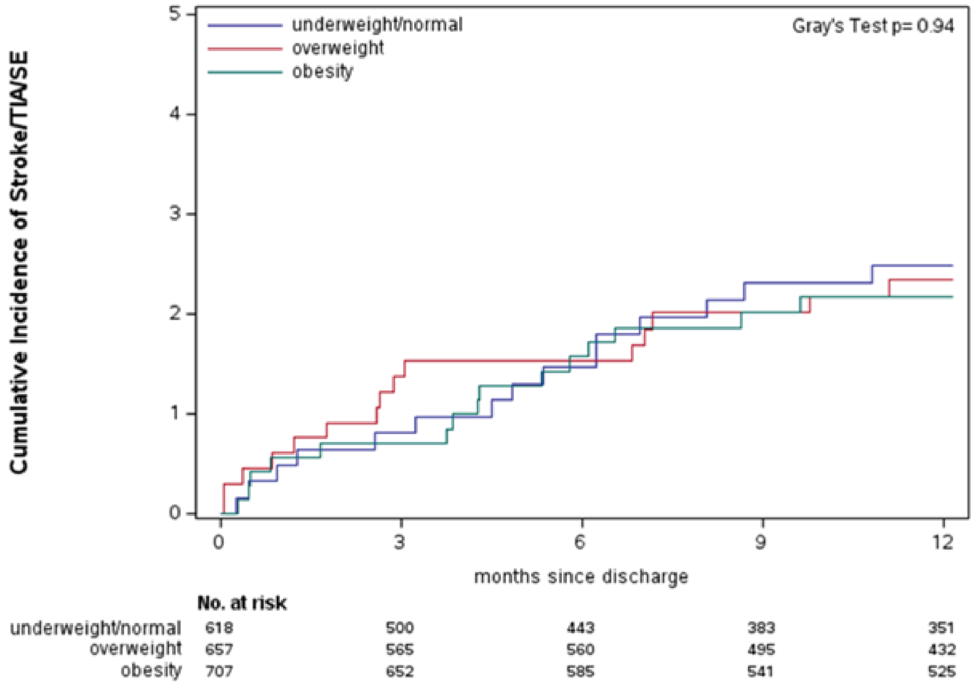

Panel C. Patients with HFpEF/HFmrEF

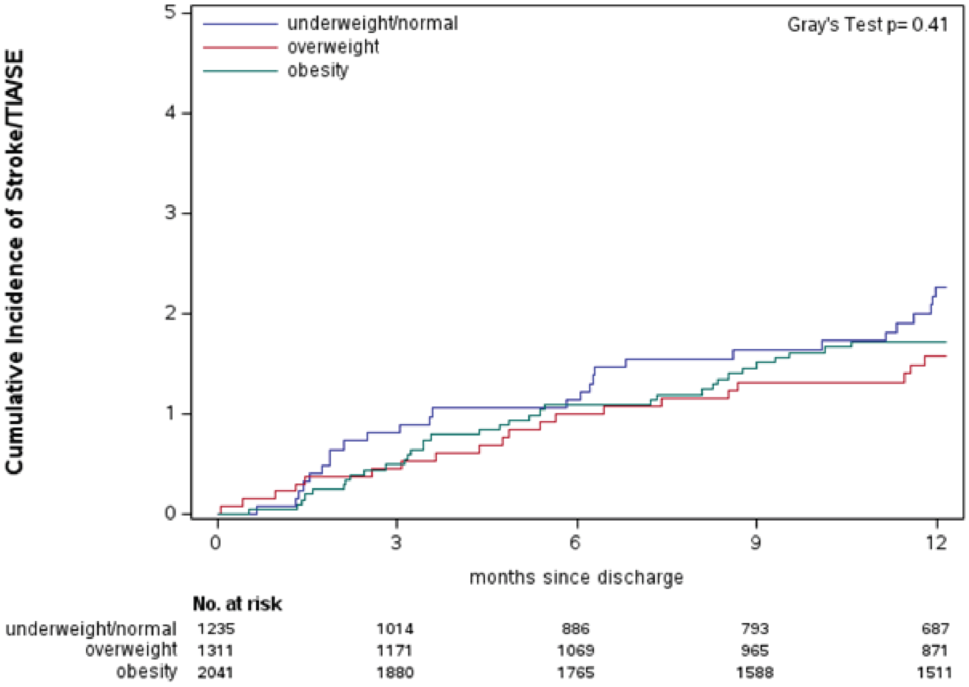

AF: atrial fibrillation; HF: heart failure; HFmrEF: heart failure with mid-range ejection fraction; HFpEF: heart failure with preserved ejection fraction; HFrEF: heart failure with reduced ejection fraction; SE: systemic embolism; TIA: transient ischemic attack

Figure S3. Cumulative incidence curves for myocardial infarction across body mass index categories in AF patients without HF (Panel A), with HFrEF (Panel B) and HFpEF/HFmrEF (Panel C)

Panel A. Patients without HF

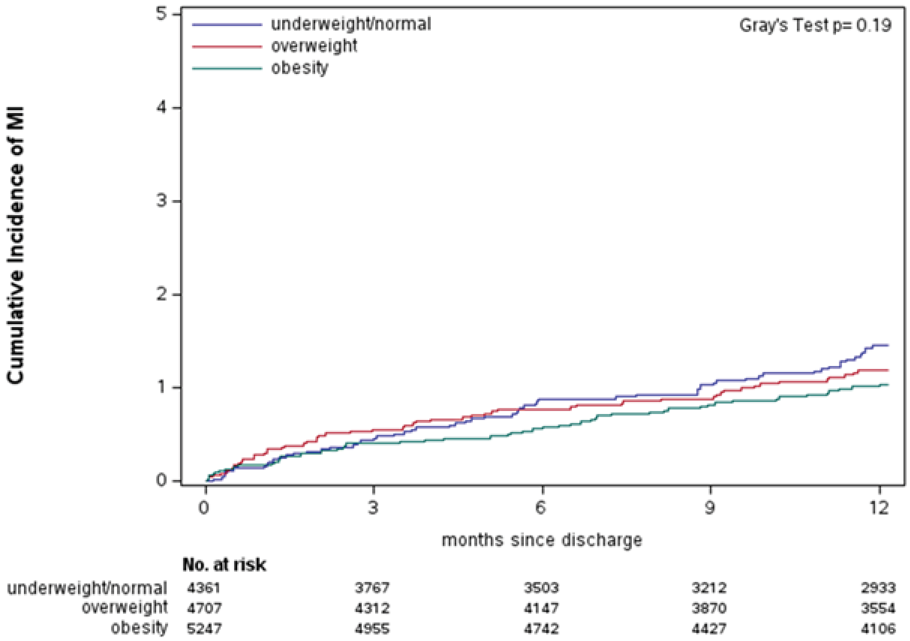

Panel B. Patients with HFrEF

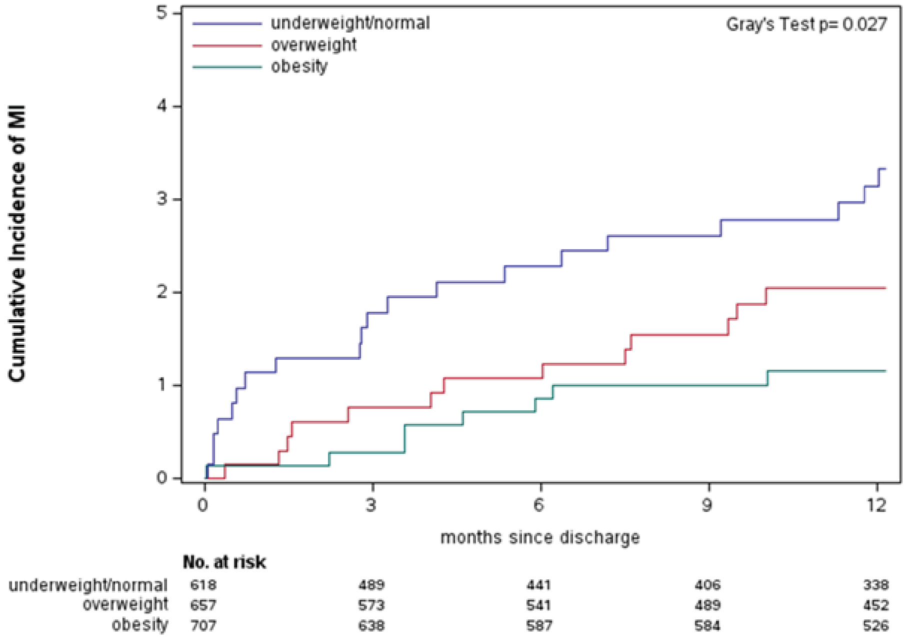

Panel C. Patients with HFpEF/HFmrEF

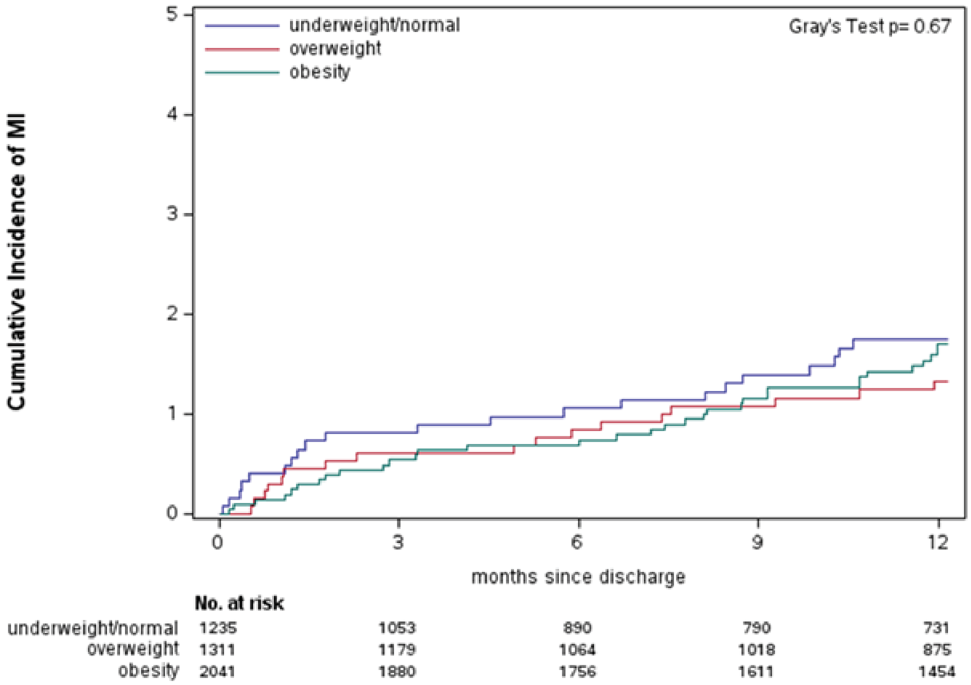

AF: atrial fibrillation; HF: heart failure; HFmrEF: heart failure with mid-range ejection fraction; HFpEF: heart failure with preserved ejection fraction; HFrEF: heart failure with reduced ejection fraction; MI: myocardial infarction
